# Forest composition change and biophysical climate feedbacks across boreal North America

**DOI:** 10.1038/s41558-023-01851-w

**Published:** 2023-10-23

**Authors:** Richard Massey, Brendan M. Rogers, Logan T. Berner, Sol Cooperdock, Michelle C. Mack, Xanthe J. Walker, Scott J. Goetz

**Affiliations:** 1 School of Informatics, Computing and Cyber Systems, Northern Arizona University, Flagstaff, AZ USA; 2 Woodwell Climate Research Center, Falmouth, MA USA; 3 Department of Atmospheric and Oceanic Sciences, University of California, Los Angeles, CA USA; 4 Center for Ecosystem Science and Society, Northern Arizona University, Flagstaff, AZ USA; 5 Department of Biological Sciences, Northern Arizona University, Flagstaff, AZ USA

**Keywords:** Attribution, Climate-change ecology, Fire ecology, Ecosystem ecology, Ecosystem ecology

## Abstract

Deciduous tree cover is expected to increase in North American boreal forests with climate warming and wildfire. This shift in composition has the potential to generate biophysical cooling via increased land surface albedo. Here we use Landsat-derived maps of continuous tree canopy cover and deciduous fractional composition to assess albedo change over recent decades. We find, on average, a small net decrease in deciduous fraction from 2000 to 2015 across boreal North America and from 1992 to 2015 across Canada, despite extensive fire disturbance that locally increased deciduous vegetation. We further find near-neutral net biophysical change in radiative forcing associated with albedo when aggregated across the domain. Thus, while there have been widespread changes in forest composition over the past several decades, the net changes in composition and associated post-fire radiative forcing have not induced systematic negative feedbacks to climate warming over the spatial and temporal scope of our study.

## Abstract (web summary)

Wildfire can lead to shifts in forest composition to more deciduous tree cover, which can have a biophysical cooling effect on climate. This study finds no net increase in deciduous cover or biophysical cooling over boreal North America in recent decades, despite widespread landscape scale change.

## Main

Boreal forest composition in North America is heavily influenced by wildfires^1,2^. In addition to self replacement, severe stand-replacing wildfires often kill needle-leaf evergreen trees and drive a shift to deciduous trees in early- to mid-succession^1–4^. This is particularly the case when fires burn deeply into organic soils, exposing mineral seedbeds, which in turn promotes the recruitment of deciduous trees^3,4^ that can dominate for decades to centuries^5–10^. Climate warming has intensified fire regimes in boreal North America over recent decades, including increases in fire frequency, severity and area burned^11–14^. This trend is expected to continue in coming decades^8–11,14^ and potentially shift North American boreal forests towards more deciduous composition^5,6,8,9,15–17^. Despite advances in understanding trajectories of fire and climate impacts on boreal forests, continental-scale understanding of changes in deciduous–evergreen composition associated with fires across the North American boreal biome is lacking. Past efforts have described local or regional fire-induced shifts in composition, but these efforts were based on field measurements alone^6,9,17^, moderate (500 m) spatial resolution satellite imagery^4^ or categorical classifications of cover types rather than continuous per-pixel cover estimates^18^. Lack of comprehensive maps of forest composition change that represent proportional cover and continuous spatial gradients at relatively fine resolution (for example, 30 m) have limited understanding of the extent, magnitudes and implications of changes in boreal forest composition associated with changing climate and fire regimes.

Energy budgets of boreal deciduous and evergreen forest stands differ substantially^19^ and therefore have different impacts on regional and global climate^4,20,21^. The higher surface reflectance and seasonally open canopies of deciduous trees lead to higher albedo than evergreen trees. Albedo often declines as boreal North American forest stands mature and transition to more evergreen cover^21,22^. Although albedo is higher year round in deciduous stands, the radiative effect is most pronounced in spring due to a combination of little to no foliage, the presence of underlying snow and relatively high insolation. These factors increase outgoing shortwave radiation in early- to mid-successional post-fire stands dominated by deciduous trees, leading to local cooling^21,23^. Because approximately one-third of Earth’s boreal forest is in North America^24^ and about half of Eurasian boreal forests are deciduous (whether dominated by conifer or broadleaf species), post-fire composition shifts in the North American boreal biome represent one of the largest potential regional energy budget feedbacks to the global climate system^20–23^.

In this Article, we assess changes in deciduous cover over recent decades across the North American boreal forest biome based on Landsat satellite observations coupled with an extensive field sample plot network. Moreover, we quantify the magnitude of albedo climate forcings associated with the observed composition changes. As part of this assessment, we developed novel maps of sub-pixel fractional deciduous cover at 30 m resolution across the entire North American boreal biome and coincident maps of tree canopy cover. These allowed us to capture gradients in composition and cover not otherwise possible with coarser resolution or categorical map products and to assess fine-scale biome-wide changes both within and outside of fire-disturbed areas, whereas most past studies have focused on burned areas alone. We further used these spatially detailed, multi-temporal maps to assess the influence of forest compositional changes on albedo-driven radiative forcing, including areas that burned between 1950 and 2018, as part of a chronosequence analysis. We find fires exerted a strong influence on forest composition that persisted for decades, but there was coincident succession of deciduous to evergreen cover, thus relatively small net biome-scale change in composition and albedo-driven radiative forcing when aggregated across the domain. Consequently, there have not yet been widespread systematic negative biophysical feedbacks to recent climate warming across boreal North America, despite highly dynamic composition changes locally and regionally.

## Continental-scale fractional deciduous cover

**Fig. 1:**
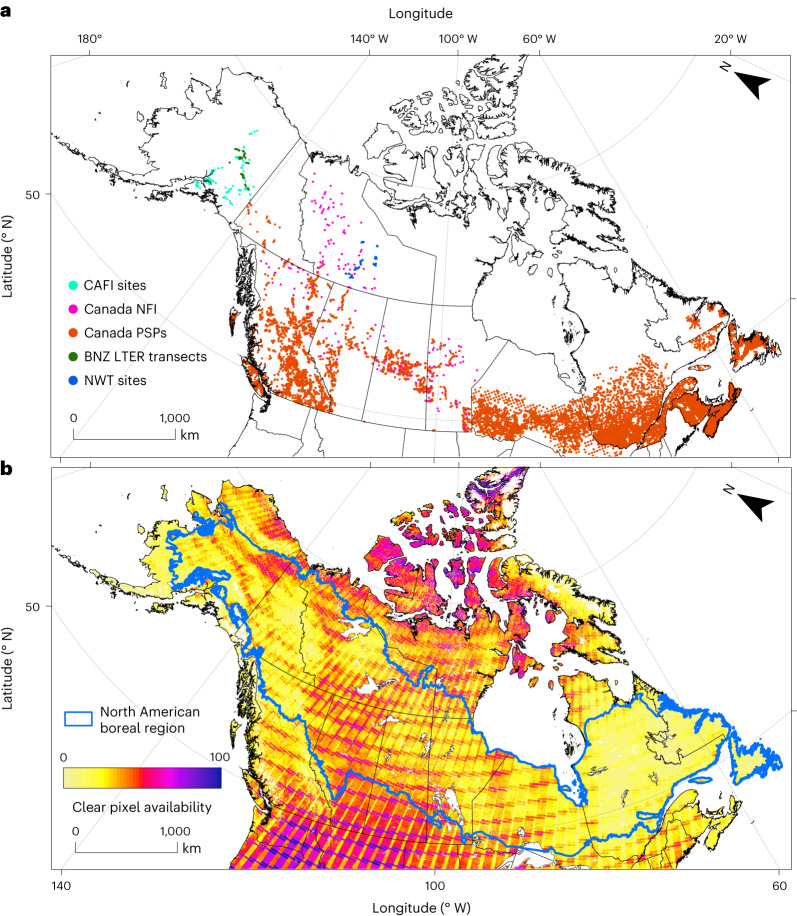
Distribution of forest inventory data and Landsat imagery used for mapping deciduous fractional cover across boreal North America. **a**, Field inventory data were from the Cooperative Alaska Forest Inventory (CAFI), Canadian National Forest Inventory (NFI), Canadian provincial permanent sample plots (PSPs), Bonanza Creek Long-Term Ecological Research (BNZ LTER) transects and the Northwest Territories (NWT). **b**, Availability of clear-sky Landsat measurements from each grid cell for the nominal year 2010. The study domain is outlined in blue.

Earth-observing satellites such as those from the Landsat series^25^ provide global observational data useful for analysing vegetation dynamics at fine spatial scales and relatively high temporal resolution^26^. We used Landsat satellite data combined with in situ forest inventory samples from multiple site networks (*n* = 27,494 plots) to develop continental-scale deciduous fractional cover and tree canopy cover maps for the North American boreal forest at multiple five-year epochs centred on 2000, 2005, 2010 and 2015. Integrating across time, in five-year intervals, was done to ensure cloud-free seasonal image mosaics needed to capture phenology and map deciduous fractional cover (Methods). For the Canadian portion of the domain, which has greater Landsat image coverage compared with Alaska, we were able to extend our mapping back to 1992. We used extensive forest inventory data that represent a robust sample of forest species and canopy conditions across broad bioclimatic gradients spanning the North American boreal domain (Fig. 1 and Supplementary Table 1). We determined deciduous fractional cover for each forest inventory plot based on the ratio of basal area of the deciduous trees compared to all trees in the plot. We then used random forest regression models^27^, trained with multi-seasonal Landsat spectral bands and indices, to predict the per-pixel deciduous fractional cover and its uncertainty from plot locations to the entire North American boreal domain. We also developed models to map tree canopy cover for the same epochs. These canopy cover maps represent the fractional areal coverage of tree canopy within each 30 m Landsat pixel. For subsequent analysis, we considered pixels with tree canopy cover fraction (proportion) greater than 0.25 (25%) as tree-dominated based on visual assessment of very high-resolution (2 m) satellite imagery at 110 randomly sampled locations stratified spatially (Supplementary Fig. 1). The combination of per-pixel deciduous fractional cover and tree canopy cover maps provide a comprehensive view of changing gradients of forest composition across the North American boreal domain over multiple time-integrated epochs.

## Deciduous forest fraction cover change

**Fig. 2:**
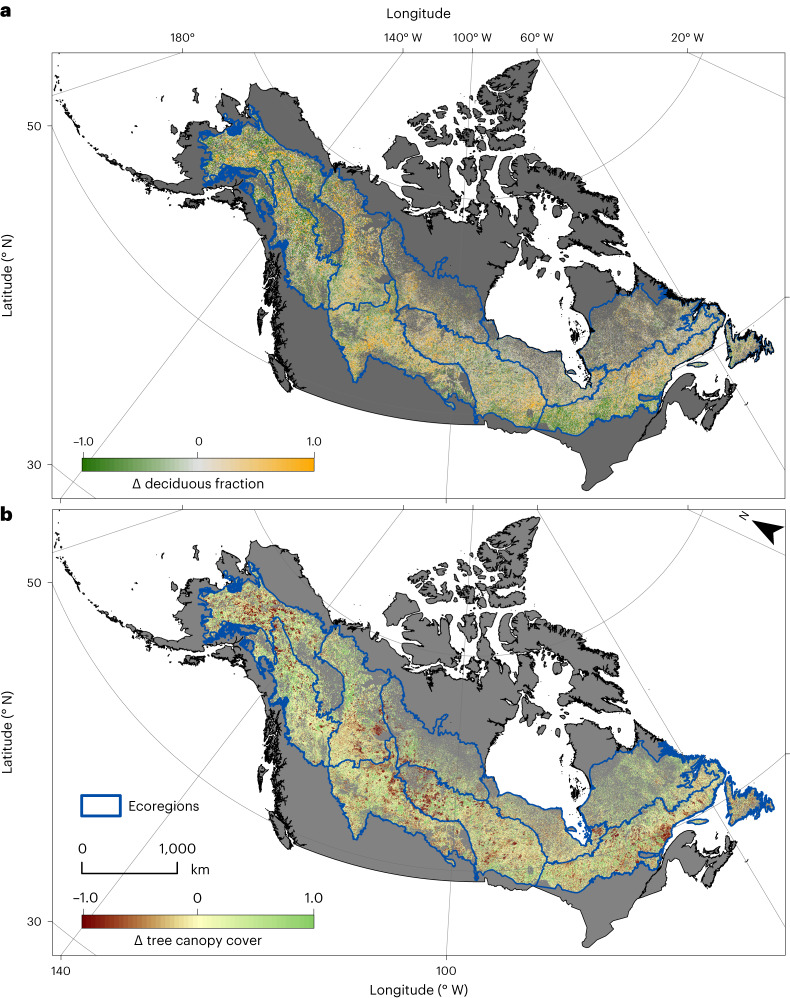
Changes in deciduous fraction and tree canopy from 2000 to 2015. **a**,**b**, Changes (Δ) in deciduous fractional cover (**a**) and tree canopy cover (**b**) from 2000 to 2015 across the North American boreal domain.

We assessed changes in forest composition across the boreal forest in North America from 2000 to 2015 and Canada from 1992 to 2015 using the deciduous fractional cover and tree canopy cover layers (Fig. 2 and Supplementary Fig. 12). It was not possible to assess pre-2000 changes in Alaska due to insufficient availability of Landsat imagery. Per-pixel deciduous fraction of the North American boreal domain showed a positive change across 166.7 million hectares (Mha; 39%) and negative change across 236.6 Mha (56%) of the 424.5-Mha boreal forest domain from 2000 to 2015 (Tables 1 and 2 and Supplementary Figs. 14–16). The remaining 5% of the boreal domain was unchanged within two decimal digit precision. When considering only pixels with statistically significant changes (Table 2 and Supplementary Fig. 15), deciduous fraction increased across 136.2 Mha (38%) and decreased across 184.3 Mha (43%) of the forested domain, whereas 19% displayed non-significant changes.

**Table 1:** Average changes in forest deciduous fraction and tree covered areas, albedo and seasonal forcing between 2000 and 2015 for areas within fire perimeters and across the North American boreal domain

	Fire perimeters			All fire perimeters	Boreal domain
	1950–1978	1978–1998	1998–2018		
Increase in deciduous fraction (10^6^ hectares)	6.63 (3.75)	16.06 (11.43)	22.33 (17.05)	47.48 (33.53)	166.71 (101.97)
Decrease in deciduous fraction (10^6^ hectares)	18.03 (12.44)	17.0 (11.56)	11.95 (7.91)	50.78 (34.12)	236.61 (150.54)
Increase in tree canopy cover (10^6^ hectares)	14.85 (11.89)	25.43 (23.4)	4.17 (3.38)	46.83 (40.32)	174.45 (132.64)
Decrease in tree canopy cover (10^6^ hectares)	9.81 (6.17)	7.63 (5.42)	30.11 (28.86)	51.43 (43.25)	228.84 (162.53)
Spring warming (W m^−^^2^)	0.181 ± 0.130	0.316 ± 0.192	0.154 ± 0.131	0.241 ± 0.171	0.073 ± 0.140
Spring cooling (W m^−^^2^)	−0.038 ± 0.175	−0.028 ± 0.184	−0.47 ± 0.232	−0.306 ± 0.095	−0.069 ± 0.197
Summer warming (W m^−^^2^)	0.035 ± 0.011	0.046 ± 0.02	0.028 ± 0.016	0.039 ± 0.020	0.013 ± 0.017
Summer cooling (W m^−^^2^)	−0.029 ± 0.026	−0.033 ± 0.023	−0.061 ± 0.028	−0.051 ± 0.030	−0.009 ± 0.027
Fall warming (W m^−^^2^)	0.08 ± 0.059	0.159 ± 0.102	0.077 ± 0.072	0.118 ± 0.089	0.034 ± 0.074
Fall cooling (W m^−^^2^)	−0.057 ± 0.082	−0.065 ± 0.087	−0.209 ± 0.109	−0.153 ± 0.112	−0.033 ± 0.091

Seasonal forcings are means ± 1 s.d. The numbers in parenthesis indicate area of statistically significant change (Supplementary Information provides specifics).

**Table 2:** Areal extent of changes in forest deciduous fraction and tree cover from 1992 to 2015 for fire perimeters and the boreal forest in Canada

Forest attribute change	Fire perimeters			All fire perimeters	Canadian boreal forest
	1950–1978	1978–1998	1998–2018		
Deciduous fraction increased	5.14 (3.78)	17.64 (15.36)	15.80 (13.77)	40.58 (34.34)	149.88 (113.11)
Deciduous fraction decreased	14.49 (12.48)	12.03 (9.63)	9.88 (7.77)	40.19 (32.81)	201.57 (155.69)
Tree cover increased	16.61 (15.29)	22.47 (20.86)	4.90 (4.14)	47.55 (43.08)	235.44 (197.17)
Tree cover decreased	3.02 (1.96)	7.20 (5.78)	20.78 (19.78)	33.22 (29.02)	116.0 (79.64)

Units of area are in Mha (10^6^ hectares). The numbers in parenthesis indicate area of significant change.

Our results from the 1992–2015 (Canada only) and 2000–2015 (boreal North America) time periods (Table 2) collectively indicate that, on average, the relative dominance of deciduous trees in the North American boreal forest slightly decreased during the past two to three decades, primarily because of succession to evergreen conifers from older fire disturbance. These findings differ from past reported increases in the extent of deciduous forest during recent decades based on plant functional type cover mapping across Alaska and Yukon territory^28^ and categorical land cover type classification across Alaska and western Canada^18^ also based on Landsat imagery (discussed below). We also found large regional differences in forest compositional change from 2000 to 2015. Average (± 1 s.d.) per-pixel deciduous fraction decreased more in Alaska (−0.058 ± 0.035) compared to western (−0.019 ± 0.012) or eastern Canada (−0.023 ± 0.015) (Supplementary Table 2). Examining changes in deciduous fraction across ecoregions^28^, the Taiga Shield West was the only ecoregion with an increase in average per-pixel deciduous fraction (+0.009 ± 0.005), whereas the largest average per-pixel decreases occurred in the Taiga Cordillera (−0.057 ± 0.041) and Boreal Cordillera (−0.055 ± 0.042).

**Fig. 3:**
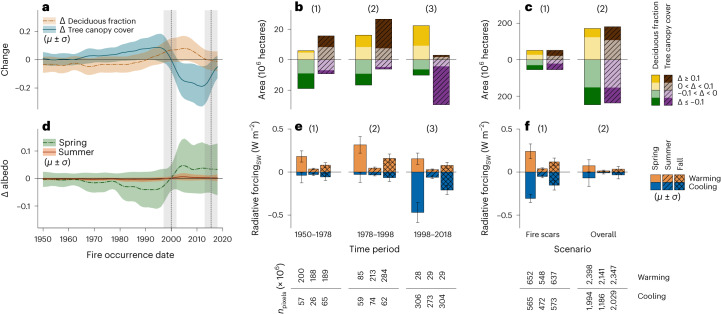
Change in deciduous fraction, tree canopy cover, albedo and seasonal forcing between 2000 and 2015. **a**, Data are presented as mean change with ± s.d. in per-pixel deciduous fraction and tree canopy cover inside fire perimeters by fire occurrence date. **b**, Area with small (under ±0.1) and large (over ±0.1) changes in deciduous fraction and tree canopy cover by three fire age classes (1) old (1950–1978), (2) intermediate (1979–1998) and (3) recent (1999–2018). **c**, Area with small (under ±0.1) and large (beyond ±0.1) changes in deciduous fraction and tree canopy cover within fire perimeters (1) and for the boreal domain (2). **d**, Mean change in spring and summer albedo with ± s.d. inside fire perimeters. **e**, Mean radiative forcing by the three seasonal and fire age classes with ± 1 s.d. bars. **f**, Mean seasonal radiative forcing with ± 1 s.d. bars within the fire perimeters (1) and for the North American boreal domain (2). Note here Δ represents change in the represented variable between 2000 and 2015. The grey dashed lines in **a** and **d** indicate the date ranges of Landsat images used in the 2000 and 2015 deciduous fraction and tree canopy cover products. The axes above and below the zero line in **b** indicate non-negative values. The number of 30 m × 30 m pixels used to calculate mean and standard deviation are indicated below the **e** and **f** plots.

To assess the role of wildfire in driving these compositional changes, we examined deciduous fraction inside burned area perimeters of varying ages (Fig. 3) obtained from Alaskan and Canadian forest fire agencies^29,30^ (Supplementary Fig. 17). For all fires that occurred after 1950, 47.5 Mha (48.3%) showed increases in deciduous fractional cover and 50.8 Mha (51.7%) showed decreases during our observational period of 2000–2015. Over the same period, tree canopy cover increased in 46.8 Mha (47.6%) and decreased in 51.4 Mha (52.3%) within fire perimeters (Table 1 and Fig. 3). Fires increased per-pixel deciduous fraction by an average of 0.09, but this was highly dependent on when the fire occurred.

We further considered three different time periods of fire history: (1) recent fires (1998–2018), (2) intermediate aged fires (1978–1998) and (3) older fires (1950–1978). The majority (75%) of the area with post-fire deciduous fraction increases occurred within burn perimeters of recent to intermediate ages, primarily due to changes from pre-fire evergreen conifers to post-fire early-successional deciduous trees and shrubs. During mid-succession (intermediate aged fires), roughly equal increases and decreases in deciduous fraction were accompanied by an overall increase in tree canopy cover (Fig. 3a,b). Older fires tended to show a continued decline in deciduous fraction with evergreen conifer succession. Overall, our results suggest that ‘relay floristics’ have been widespread after fire^20^, with deciduous fraction initially increasing after fire but then progressively declining as evergreen conifers establish dominance.

## Effect of composition changes on albedo and radiative forcing

Forest composition played a major role in changing surface albedo and associated radiative forcings. Cooling effects associated with deciduous trees tend to be relatively small in summer but more prominent in spring and fall due to increased snow exposure under deciduous canopies^21^. We determined median seasonal albedo using daily ‘blue-sky’ albedo derived from the MODIS satellites^22^ combined with radiative forcing kernels from the Community Earth System Model (CESM)^31^ (below) and then trained random forest models to predict seasonal albedo based on deciduous fraction and tree canopy as predictors. We used the differences between predicted 2000 and 2015 seasonal albedo composite layers to derive seasonal shortwave radiative forcing for spring, summer and fall. We did not include winter albedo in this study because winter forcings are smaller compared to other seasons at these latitudes^21^ and, crucially, the quality of albedo data from remote sensing tends to be unreliable due to short daylengths in the winter months^23,32–34^. We note, however, that fire-induced changes in winter albedo and radiative forcing tend to be in the same direction as changes in spring, summer and fall^21^.

We assessed how changes in deciduous fraction and tree canopy cover between epochs impacted the seasonal albedo and associated radiative forcing. Specifically, we used radiative forcing kernels for albedo^31^ from the Community Atmosphere Model 5 (CAM5)^35^ in the CESM to calculate seasonal forcing. To do so, we first estimated a change in albedo between the two time periods and then multiplied these pixel-level changes by the spatially explicit kernels, which were spatially downscaled to match the 30 m resolution of our canopy composition and cover products. In addition to domain-wide albedo forcing for tree-dominated pixels, we also assessed forest fire-induced composition alterations within burned area perimeters. Our random forest models explained significant variability in surface albedo with cross-validated *R*^2^ values of 0.36, 0.44 and 0.29 for spring, summer and fall seasons, respectively (*p* < 0.05 for all three seasons). Spring and fall season models had higher explanatory power from tree canopy cover, whereas deciduous fraction explained most of the variability in summer (Supplementary Fig. 2), which is consistent with the phenomenon of underlying snow dominating the albedo signal in non-summer months and tree species composition dominating the signal in summer months^21^.

**Fig. 4:**
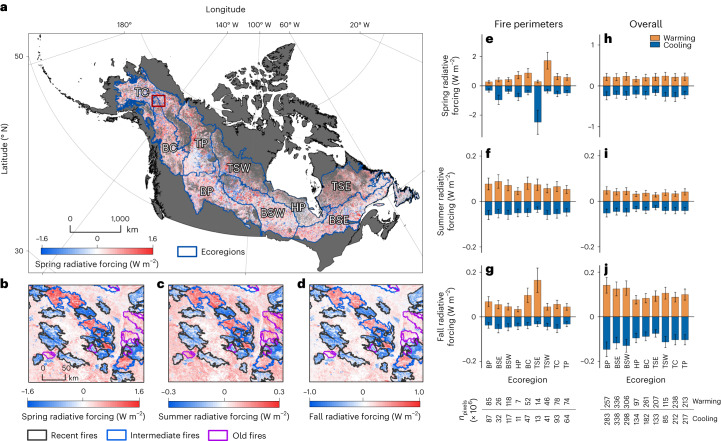
Radiative forcing across boreal domain. **a**–**d**, Per-pixel spring forcing for boreal domain in North America between 2000 and 2015 (**a**) with changes in spring (**b**), summer (**c**) and fall (**d**) radiative forcing inside recent (1999–2018), intermediate (1979–1998) and older (1950–1978) fire perimeters in a small portion of interior Alaska (marked with red boundary in the change map). **e**–**j**, Average (±1 s.d.) positive and negative forcing within fire perimeters (**e**–**g**) and overall (**h**–**j**) for each ecoregion in the boreal domain. Ecoregions include the Taiga Cordillera (TC), Taiga Plain (TP), Boreal Plain (BP), Boreal Cordillera (BC), Taiga Shield West (TSW), Boreal Shield West (BSW), Hudson Plain (HP), Taiga Shield East (TSE) and Boreal Shield East (BSE). Note we only included fire perimeters with cumulative areas greater than 10,000 hectares in panels **b**–**d**. Number of 30 m × 30 m pixels in each ecoregion used to calculate mean and standard deviation for **e**–**j** are indicated below the **g** and **j** plots.

Across boreal North America, changes in deciduous fraction and tree cover canopy from 2000 to 2015 generated a small net biophysical warming of 0.004 ± 0.002 W m^−2^ in spring, 0.004 ± 0.001 W m^−2^ in summer and 0.001 ± 0.001 W m^−2^ in fall, with an average of 0.003 ± 0.001 W m^−2^ across non-winter months (Table 1). These overall changes were the net result of regional heterogeneity in deciduous and evergreen forest changes due to the interplay between fire disturbance history, climate and other factors (Fig. 4). Although albedo forcings from fires were locally strong within burn perimeters, negative forcings from recent burned areas and positive forcings from older burned areas largely cancelled each other out, resulting in relatively small net domain-wide outcomes (Fig. 3f and Fig. 4b–d). Most recent fires (1998–2018) generated large negative shortwave radiative forcings (that is, cooling) across all the non-winter seasons due to increases in surface albedo between the 2000 and 2015 epochs, whereas burn perimeters of intermediate age (1979–1998) generated mostly positive seasonal forcings (that is, warming; Fig. 3e). In mid-successional forests, the overall net change in deciduous canopy cover was relatively low but increasing tree cover generated a biophysical warming effect. Similar patterns were observed for older fires (1950–1978), albeit with lower magnitude due to slower changes in tree cover and forest composition in older stands. The overall radiative forcing inside burned area perimeters averaged −0.058 ± 0.012 W m^−2^ for spring, −0.012 ± 0.005 W m^−2^ for summer and −0.036 ± 0.010 W m^−2^ for fall, leading to a relatively small cooling effect across non-winter months (Fig. 3f). This effect was amplified in the Taiga Shield East ecoregion due to large fire years in 1989 and 2013 (Supplementary Fig. 18).

## Implications for forest–fire–climate feedbacks

Our results indicate that despite extensive shifts in forest composition over recent decades, there was little net change in the overall proportion of deciduous and evergreen cover across the North American boreal forest and thus nearly neutral non-winter net radiative forcings over the same period. Similarly, fires since 1950 contributed relatively little to net changes in forest composition, with increasing deciduous cover and biophysical cooling from recent fires largely being offset by decreasing deciduous cover and biophysical warming from intermediate aged and older fires.

While not specifically accounting for understory shrubs, our analysis showed little net change in deciduous fractional cover across the North American boreal forest. This was unexpected given our current understanding of how recent changes in climate and fire regimes affect forest composition. A large body of literature indicates deciduous species are more competitive than evergreen conifers in severely burned areas, a phenomenon exacerbated by the warmer and drier climate Alaska and western Canada have experienced over the previous 30–40 years (refs. ^1,2,7,8^). Permafrost thaw influences canopy density via soil water availability and in areas where drainage is facilitated can lead to more rapid mineralization and increases in plant-available nutrients, which are expected to favour fast-growing deciduous species over conifers where conditions are otherwise favourable^16^. As a result, some studies have predicted increases in deciduous cover during the twenty-first century in Alaska^15,16^ and associated decreases in landscape flammability^10,21^. While such large-scale increases in deciduous trees may still occur, our results show there has yet to be a systematic shift to greater deciduous cover across the North American boreal forest.

Within burned areas, our results also show increases in evergreen dominance in older fire scars counteract increases in deciduous cover from recent fires. Fires that occurred before 1992 showed a small mean decrease in deciduous fraction between 2000 and 2015, consistent with succession towards more evergreen conifers. This is also consistent with increasing evergreen cover outside mapped fire scars, as our analysis included fire perimeters as old as 1950 and gradual successional changes can occur even 100 years post-fire^36^. Decades of fire suppression around an expanding wildland urban interface in Alaska and Canada may have also played some role in increasing evergreen cover^37,38^. Other potential factors may include the impacts of drought and insect outbreaks on deciduous tree species^39–41^, an inability for deciduous trees to regenerate in thick organic soils without severe fires and the role of jack (*Pinus banksiana*) and lodgepole (*Pinus contorta*) pine in post-fire succession throughout much of Canada^41–43^.

Similar processes may be playing out in Eurasian boreal forests, which comprise most of the boreal forest biome^22^. Although the biophysical trends we observed after fires are consistent with past work in both boreal North America and Eurasia, the magnitudes of albedo and forcing changes differ substantially by continent, region and analysis technique^22,23,44,45^. Eurasian boreal forests have a much higher fraction of non-stand-replacing surface fires and prevalence of deciduous needle-leaf conifers (*Larix spp*.), resulting in post-fire deciduous fraction, canopy cover and albedo change across large areas that are likely to be driven more by stand density and regeneration success than deciduous broadleaf–conifer competition^4,6,22^.

Future changes in fire frequency, timing and severity could alter these patterns and successional cycles^4,6,9^. For example, with fires in the boreal domain projected to increase with warming climate, the successional pathways of post-fire deciduous species such as quaking aspen (*Populus tremuloides*) being replaced by evergreen conifers such as black spruce (*Picea mariana*), white spruce (*Picea glauca*) or jack pine during intermediate and older fire ages may occur less frequently, thereby prolonging the persistence of deciduous forest cover^9^. Moreover, severe fires and/or more frequent re-burning decrease soil organic depth and expose greater areas of mineral soil, allowing deciduous trees to rapidly regenerate. Deciduous expansion can also be facilitated by permafrost thaw, associated availability of soil nutrients and increased prevalence of deciduous trees across the landscape that aid post-fire regeneration through seed dispersal, belowground bud banks or root suckering^17^. Post-fire regional cooling is also expected to increase in the future with an intensifying fire regime^19^, although earlier snowmelt will probably decrease the magnitude of cooling^23^. This effect is expected to be most pronounced in areas with high evergreen forest cover and closed canopies. The fact that we did not, on balance, observe such changes across the domain during the past 20–30 years highlights the potential for other mechanisms influencing conifer–deciduous dominance. For instance, some conifer species (for example, black spruce) may be replaced post-fire by other evergreen conifers (for example, jack and lodgepole pine) that are more tolerant of drought conditions on mineral soils.

## Methods

### Deciduous fractional layers

We developed deciduous fraction layers using random forest regression models in a supervised-learning approach (Supplementary Fig. 3). We used satellite data from Landsat 5 TM, Landsat 7 ETM+ and Landsat 8 OLI sensors to derive deciduous fractional cover and tree canopy cover layers at 30 m spatial resolution. Whereas the Landsat datasets are publicly available to download through the US Geological Survey, we used Google Earth Engine^46^ (GEE) to pre-process these datasets via GEE Python application programming interface. We used Landsat 5, 7 and 8 Collection 1 surface reflectance datasets for years 1987–1997, 1998–2002, 2003–2007, 2008–2012 and 2013–2018 to derive normalized difference vegetation index (NDVI)-based median-value image composites for nominal years 1992, 2000, 2005, 2010 and 2015, respectively. We used NDVI to derive median-value composites for spectral bands, which were then used to calculate other indices. These image composites were prepared for early spring, mid-summer and mid- to late-fall seasons to identify key differences in deciduous and evergreen green-up amplitudes (Supplementary Table 3 and Supplementary Fig. 4). In addition to Landsat surface reflectance bands, we added several vegetation indices derived from Landsat bands as additional features for the random forest regression model: (1) normalized difference vegetation index (NDVI), (2) normalized difference wetness index, (3) soil-adjusted vegetation index, (4) variable atmospherically resistant index and (5) enhanced vegetation index (EVI). With the three seasonal composites and addition of slope, elevation and aspect as topographical variables from the GMTED2010 dataset^47^, the image composite layer stack consisted of 36 bands or features (Supplementary Fig. 5).

We obtained ground samples for modelling deciduous fraction from (1) the Cooperative Alaska Forest Inventory (CAFI)^48^; (2) the Bonanza Creek Long-Term Ecological Research (BNZ LTER) samples in Alaska^49^; (3) repeatedly measured permanent sample plots (PSPs)^50^ from the Canadian provinces of Yukon, Northwest Territories, British Columbia, Alberta, Saskatchewan, Manitoba and Ontario; (4) ground sample plots from Northwest Territories^51^ and (5) Canadian National Forest Inventory^52,53^ plot data for sites across Canada (Fig. 1 and Supplementary Table 1). These ground samples include species-specific basal areas at each site. We converted the species-specific basal areas to deciduous fraction by taking the ratio of deciduous tree basal area to total tree basal area to be used as the response variable in random forest^27^ regression models.

We used the *RandomForestRegressor* module in the scikit-learn Python package as our random forest regression model^45^. We divided the sample locations geographically into zones: (1) east and (2) west (Supplementary Fig. 6) due to differences in spatial density of ground samples, significantly different model outputs and large variations in importance of input features in the random forest regression models. In addition to geographically distinct random forest models, we trained two temporally distinct random forest models for each zone: (1) a three-season model, using spring, summer and fall seasonal composites and (2) a one-season model, using summer seasonal image composite. The one-season model used summer season image composite as input and was applied to pixels where at least one of the spring, summer and fall models were not available, resulting in gaps in the three-season model output. The three-season model was used for 83.6 to 91.2% of the boreal domain area across the nominal years (Supplementary Table 9). To reduce bias in the random forest models due to data imbalance from the large number of evergreen sample plots (<5% deciduous fraction) and deciduous sample plots (>95% deciduous fraction), we resampled each zone’s sample set by binned under-sampling to create a near uniform distribution of the response variable–deciduous fraction (Supplementary Fig. 7). We used 70% of the samples for each model for training and 30% for validation. The random forest models were then parameterized using a multi-dimensional grid search approach with number of trees (*n_estimators*), maximum number of features per tree (*max_features*), minimum number of samples per split node per tree (*min_samp_split*) and minimum number of samples at each leaf node in a tree (*min_samp_leaf*) as parameters (Supplementary Table 4). For each combination of the parameters, we parameterized 1,000 fivefold cross-validated model runs up to a total of 30 million models to perform a grid search for parameter sets resulting in the highest mean cross-validated *r*^2^ value, low root mean-squared error (RMSE) and low standard deviation in RMSE (Supplementary Table 4). This grid search for parameters was performed using the training samples. The random forest regression models were then applied to tiled Landsat image composites. Landsat-derived seasonal composite bands that are highly correlated (absolute value of correlation coefficient >0.95; Supplementary Fig. 8) were not used in random forest models as inputs. We used variable importance plots to assess and validate bands used in the random forest models (Supplementary Fig. 9). We also used one standard deviation of all samples in output leaves across all the decision trees in the random forest regression model as a measure of uncertainty for deciduous fractional cover layers. Additionally, the samples not used in training the model were used to provide an estimate of variance explained by the random forest model using adjusted *r*^2^ value and model performance using RMSE value (Supplementary Table 4). Spatial autocorrelation effects in validation samples were reduced by resampling all the validation plot locations via gridding and using a minimum distance threshold of 1 km. We also compared the deciduous fraction layers with previous work that produced categorical maps of forest cover resulting in a similar consensus across the three products (Supplementary Fig. 10).

### Tree canopy cover layers

The tree canopy cover layers were developed using a random forest modelling framework similar to deciduous fraction (Supplementary Fig. 3). The training samples were selected in a stratified random manner, with a stratum for each tree canopy cover level that was extracted from the global tree cover data for 2010^26^. We then used the tree canopy cover sample locations to extract band values from the three-season 36-band image layer stack. Similar to our deciduous fraction modelling approach, we used the *RandomForestRegressor* module in the scikit-learn Python package as our random forest regression model to derive tree canopy cover layers. We also used a one-season model to fill in gaps from the three-season model. We did not divide the study area into different zones for tree canopy cover modelling. We extrapolated global tree cover data for 2010 to nominal years 1992, 2000, 2005, 2010 and 2015 in North American boreal region using the random forest models. We used 30% of the ground samples that were selected to build the models exclusively for model validation for tree canopy cover layers. The parameterization of the model was performed as in the deciduous fractional layer approach (Supplementary Table 4). To determine uncertainty in tree canopy cover value we used one standard deviation from all samples in output leaves across all the decision trees in the random forest regression model.

### Assessing change and albedo-based radiative forcing

We used spring (March–May), summer (June–August) and fall (September–November) season image composites of the blue-sky albedo product for 2000, 2005, 2010 and 2015 to assess changes in albedo along with the respective deciduous fraction and tree canopy cover products. For each seasonal mean-value composite, we used a threshold of solar zenith angle <70° to determine valid data. Areas with low post-fire tree cover (<25%) were included in our analyses if they initially had higher (>25%) tree cover values before burning. For example, we included pixels in our analysis from 2000 to 2015 that had tree cover reduced from 75% before year 2000 to 10 or 20% after 2000 due to fire mortality. To examine the strength of relationship between albedo, deciduous fractional cover and tree canopy cover, we modelled albedo for spring (March–May), summer (June–August) and fall (September–November) using albedo data from 2000, 2005 and 2010 in a random forest supervised-learning approach (Supplementary Fig. 3). The training samples were derived by compositing daily blue-sky albedo layers to seasonal median values and resampling from 500 m spatial resolution to 30 m using bicubic convolution. We extracted stratified random samples within each 0.1 albedo interval to train the random forest regression model. We parameterized the random forest model by optimizing *r*^2^ and RMSE values using a multi-dimensional grid search approach to determine the best set of random forest regression parameters: *n_estimators*, *max_features*, *min_samp_split* and *min_samp_leaf* (Supplementary Table 5). We multiplied the difference in our modelled albedo between the two epochs 2015 and 2000 by the ‘all-sky’ CESM-CAM5 albedo kernel^31^ to compute radiative forcing. We extracted mean values of positive and negative change in deciduous fraction, tree canopy cover, blue-sky albedo composites and radiative forcing to assess changes inside fire perimeters and across the entire domain. We then used fire boundaries obtained from Alaska Large Fire Database^29^ and Canadian National Fire Database^30^ for fires that occurred between 1950 and 2018 to assess change. We used >500,000 stratified random samples to assess the validity of deciduous canopy layers by examining the relationships between deciduous fraction, tree canopy cover and uncertainty (Supplementary Fig. 11).

### Caveats

Although our deciduous fractional cover and tree canopy layers capture the variation in tree-based forest composition across the boreal domain (Supplementary Fig. 12), they do not specifically consider the understory component of the boreal forest because the forest inventories did not consider trees less than 1.5 m height. This understory vegetation component may contribute to the unexplained variance in the deciduous fraction model and impact inferred patterns of post-fire succession. Another limitation of this analysis is the dependency on multi-spectral data to extrapolate deciduous fraction and tree canopy cover across the boreal domain. Because we trained the regression model on the spectral reflectance of tree-based deciduous fraction, there may be locations where the spectral reflectance of shrubs and other vegetation types resembles that of the treed components. Whereas we used tree canopy cover as a masking layer to only include treed forest in our study, locations with high shrub density may be modelled as having higher tree canopy cover than the threshold, making them difficult to exclude. However, we note that the deciduous signal seen in canopy dominants could translate to vegetation <1.5 m, particularly in early succession with small deciduous trees and shrubs. Therefore, even though we have only trees >1.5 m in our training database, the random forest model will return a high deciduous fraction value for a pixel even if it contains smaller trees with a spectral signature similar to deciduous canopy dominants.

Per-pixel deciduous fraction does not depict vegetation cover but rather the dominance of deciduous versus evergreen vegetation, independent of the per-pixel canopy cover. While 30 m spatial resolution is fine enough to represent homogeneous vegetation, this pixel size may include mixed vegetation types such as deciduous shrubs and evergreen trees. Such mixing can in areas of lower tree canopy cover result in some pixels having deciduous fraction greater than the percentage of tree cover (for example, low tree cover but all deciduous).

In this study we did not differentiate between deciduous broadleaf versus deciduous needle-leaf. Deciduous needle-leaf trees are not widespread in boreal North America. Rogers et al.^22^ estimate larch cover <0.5% of boreal forests in North America and account for 0.01% of fires. Further, due to lack of remote sensing data for an entire fire cycle, which is typically of the order of 80–150 years (ref. ^20^), we analysed variations in older fire scars across a time period of 15 years to assess changes in forest composition and forcing in early- to mid-successional post-fire periods. We did not analyse fires in the late-successional period in this study due to lack of reliable fire perimeter data from fires that occurred 80–150 years ago. However, our assessment of changes in forest composition and forcing for the entire North American boreal domain provides an indication of variations in such late-successional fire perimeters.

The fire perimeters used in this study were acquired from the respective state agencies and may not represent burned areas accurately in the early decades of analysis^23^, nor account for unburned islands or variations in fire intensity and burn severity, which are known to influence post-fire regeneration success and composition^21^. Our exclusion of pixels with <25% canopy cover probably excluded some otherwise forested pixels within fire perimeters from the 1980s and 1990s. Because monthly albedo tends to increase for roughly ten years after a fire and then gradually decrease^23^, it is unclear what effect these omissions have on our albedo analysis. These exclusions also probably minimally affect our estimates of changes in deciduous fraction, given fires before 1990 tended to show decreases in deciduous fraction between 2000 and 2015, and fires after 1990 showed increases (Fig. 3). However, they may impact our assessment of overall changes in tree cover (Table 1), given fires during this period tended to exhibit strong increases in tree canopy cover between 2000 and 2015.

In choosing seasonal time periods for our albedo analysis, we used fixed months that may or may not correspond to relevant physical and environmental seasonal changes for any given pixel in any given year. However, this choice facilitated modelling and comparisons across space and time. Finally, we were unable to account for potentially earlier snowmelt in spring due to warming during the analysis periods, which would otherwise lead to more biophysical warming^22^.

## Online content

Any methods, additional references, Nature Portfolio reporting summaries, source data, extended data, supplementary information, acknowledgements, peer review information; details of author contributions and competing interests; and statements of data and code availability are available at 10.1038/s41558-023-01851-w.

### Supplementary information

Supplementary Information Supplementary methods, Tables 1–9 and Figs. 1–18.

## Data Availability

The deciduous fraction composition and tree cover raster datasets generated in this study are publicly available through the Oak Ridge National Laboratory Distributed Active Archive System (ORNL DAAC) at 10.3334/ORNLDAAC/2296.

## Code availability

We used Python 3.7.5 programming language for all workflows in this analysis. Data extraction from the raster layers was performed using custom packages and codes written in Python programming language. All statistical and machine learning analyses were performed using Python packages numpy 1.15.0, scipy 1.2.0 and scikit-learn 0.23.2. We developed custom Python packages geosoup and geosoupML to process, extract and manipulate geospatial data on multiple compute platforms and to use random forest and multiple linear regressor to extrapolate deciduous fraction and tree canopy cover from point samples over the entire boreal domain in our workflow and codes. The custom Python packages are available at https://github.com/masseyr/geosoup and https://github.com/masseyr/geosoupML, respectively. We also developed Python package eehelper primarily to extract and manipulate geospatial data using GEE. This Python package is available at https://github.com/masseyr/eehelper. The Python codes and bash scripts used in our work are available at https://github.com/GEODE-Lab/DeciduousFraction.
