# Supplementary material for: Forest composition change and biophysical climate feedbacks across boreal North America
Source: Nat Clim Chang. 2023 Oct 23;13(12):1368–75. doi: 10.1038/s41558-023-01851-w (PMC10695824; doi:10.1038/s41558-023-01851-w)
Supplement: Supplementary file 1 — Supplementary methods, Tables 1–9 and Figs. 1–18. [file 41558_2023_1851_MOESM1_ESM.pdf]

nature climate change

Article

https://doi.org/10.1038/s41558-023-01851-w

Forest composition change and biophysical climate feedbacks across boreal North America

In the format provided by the authors and unedited

Supplemental Material

Contents

Appendix 1: Methods (continued) 3
1.1 Random forest model validation 3
1.2 Random forest model performance 3
1.3 Random forest model uncertainty 4
1.4 Random forest model comparison with linear regression 5
1.5 Tree canopy cover threshold 5
1.6 Landsat data 6
1.7 Resolution overlap with ground samples 7
1.8 Deciduous fraction and tree canopy cover 8
1.9 Ground sample selection 9
1.10 Method design 10
1.11 Blue-sky albedo 10
1.12 Significant change 11
Appendix 2: Tables 13
Supplementary table 1 13
Supplementary table 2 14
Supplementary table 3 15
Supplementary table 4 16
Supplementary table 5 17
Supplementary table 6 18
Supplementary table 7 19
Supplementary table 8 20
Supplementary table 9 21
Appendix 3: Figures 22
Supplementary figure 1 22
Supplementary figure 2 23
Supplementary figure 3 24
Supplementary figure 4 25
Supplementary figure 5 26

Supplementary figure 6 27
Supplementary figure 7 28
Supplementary figure 8 29
Supplementary figure 9 30
Supplementary figure 10 31
Supplementary figure 11 32
Supplementary figure 12 33
Supplementary figure 13 34
Supplementary figure 14 35
Supplementary figure 15 36
Supplementary figure 16 37
Supplementary figure 17 38
Supplementary figure 18 39
References 40

Appendix 1: Methods (continued)

1.1 Random forest model validation

Samples for both deciduous fraction and tree canopy cover were divided into training samples (70%) and validation samples (30%). The validation samples were not used for any part of model development. For deciduous fraction, we performed autocorrelation correction and statistical binning to reduce bias prior to splitting the sample set into eastern and western. Eastern and western sample sets were further divided into training and validation sample sets. We then reduced highly correlated (>0.95 correlation coefficient) features. We used a pair-wise elimination process to eliminate one of the features from a highly correlated pair of features. In this process, we evaluated a random forest model constructed using all training samples and default parameters and eliminated the feature with lower variable importance score from the feature pair. Each training sample set was further divided into 5 folds for cross validation of model parameters and performance. We used a brute-force search to obtain the parameters of the random forest model with the mean and standard deviation of each 5-fold cross-validation run as the evaluation metric.

1.2 Random forest model performance

We evaluated the random forest models using r-squared and RMSE values. The random forest models trained with 3-season data had a higher r-squared value than those trained with 1 season data (supplementary table 5). We found that in addition to summer surface reflectance and derived indices, fall and spring features add significantly to the model. Further, the models trained with western Canada and Alaska samples performed better in comparison to the models

trained with eastern Canadian sample plots. Across the boreal domain, 19 % of the area used 1-season random forest regression model. Most of this area was concentrated in the northern parts of Alaska and Canada.

Alaska and western Canada had summer NIR as the most important variable for both 1-season and 3-season deciduous fraction random forest models (supplementary figure 9). In the eastern Canadian region, the 1-season model had summer SWIR1 as the most important variable with spring SWIR2 as the most important variable for the 3-season model. In both the models, derived indices (summer NDVI and NDWI) generally ranked higher in the variable importance charts as compared to spectral and topographical bands.

We observed a similar trend for random forest regression for tree canopy cover where the 3-season model performed better than the 1-season model (supplementary table 5). We did not split the boreal domain geographically for tree canopy cover samples since the sample density was spatially uniform. We observed no significant spatial variations between uncertainty values in the eastern and western regions of the boreal domain. Post-validation, we found no significant spatial variations in differences between the validation samples and the predicted tree canopy cover values.

1.3 Random forest model uncertainty

The prediction uncertainty of the random forest model provides an estimate of variability in prediction due to reasonable changes in input spectral and topographical values. With lower or higher output values in deciduous fraction or tree canopy cover, the uncertainty is typically lower. With output values in the middle of output range (0 – 1 for deciduous and 0 – 1 for tree

canopy cover), the uncertainty values increase as more decision trees diverge in their outputs in the random forest. For both deciduous fraction and tree canopy cover, we used variation in individual tree outputs as a measure of model uncertainty. The collection of individual tree outputs in a random forest provides a complete conditional distribution for the response variable. We used 1 standard deviation of all the tree outputs in a random forest model as a measure of uncertainty for deciduous fraction and tree canopy cover values. The uncertainty values typically ranged from 0 – 0.35 for deciduous fraction and 0 – 0.4 for tree canopy cover.

1.4 Random forest model comparison with linear regression

We compared random forest model outputs with corresponding linear regression models. In all cases, random forest model performed better than the multiple linear regression model. The gain in r-squared value was greater than 24% for 3-season model and greater than 19% for 1-season model (Supplementary table 5). The sample set we used for random forest regression model training and validation ranged from 0 to 1 in deciduous fraction. Since the entire sample output range was covered, there was no extrapolation required outside the sample set values. The performance of random forest regression was noticeably better due to its ability to model non-linear relationships between deciduous fraction and input spectral and topographical features as compared to multiple linear regression.

1.5 Tree canopy cover threshold

We used a threshold of 25% to differentiate tree covered and non-tree covered pixels. While this definition of tree cover threshold is somewhat conservative as compared with other studies (e.g., 20%)[1], we found our visual assessment of 110 high-resolution satellite images agreed well with

this 25% threshold. The locations for visual assessment of the high-resolution imagery were chosen by identifying <25% tree cover locations with relatively low variations across forested areas. We used high-resolution imagery mosaics provided by ESRI from GeoEye-1 and WorldView-3 satellite data at 1.65m spatial resolution and a tree cover product[2] as a mask layer to identify such areas to randomly sample spatially. Because our tree canopy cover products are derived from an existing product[2] we used the same definition of tree height at 5m. However, this is different from tree height (1.5m) used in PSP data for our deciduous fraction product.

1.6 Landsat data

We included approximately 5 years of data for each nominal year as there was sufficient data available except for 1992. Nominal year 1992 used 10 years of Landsat 5 TM data to account for low annual data density across Alaska due to absence of ground receiving stations. Therefore, while we analyzed the difference in deciduous fraction, tree canopy cover, and albedo for the entire domain using years 2000 and 2015, we were able to analyze changes in the Alaskan portion of the boreal domain from 1992 – 2015. The nominal years 2005 and 2010 were used to produce additional deciduous fraction and tree canopy cover layers, primarily to maintain continuity in the data products and also to train random forest models to predict surface albedo across all years.

We used Landsat “pixel_qa” band available Collection 1 SR data to only include pixels that were identified clear from snow, cloud, or cloud shadows. We then used NDVI-based image composites for synthesizing seamless Landsat mosaics across Alaska and Canada since there were significant parts of the boreal domain that had large parts of a year covered in snow or ice. This reduced the number of “clear” pixels for such regions. The average NDVI values for pixels

in such regions were therefore greatly influenced by non-clear observations. We chose median NDVI composites to help mitigate such effects of excessive snow or ice presence or low clear observations in a pixel for any given year. These seasonally representative values provided sufficient variability across all spectral bands and indices to derive a continuous distribution of deciduous fractional value from 0 to 1.

We used a set of coefficients and linear regression models[3, 4] to correct spectral bands of Landsat 8 OLI and 5 TM to make them consistent with Landsat 7 ETM+ surface reflectance. In our analysis, it was critical to consider and correct sensor differences to train the random forest models with data from multiple years and use them to compute deciduous fraction and tree canopy cover layers. Since all spectral bands were calibrated between sensors, NDVI and other indices were assumed to be sufficiently calibrated across the sensors allowing us to compute NDVI-based image composites.

1.7 Resolution overlap with ground samples

The PSP ground sample plot sizes ranged between 14.2m × 14.2m and 44.7m × 44.7m. For the majority of plots (>97% of all samples), we did not have the plot boundary polygons as they are not digitized by the Canadian Forest Management Branch. For such point locations, we extracted all 9 pixels from the prepared Landsat composites in a 3 × 3 grid centered on the sample location. The final sample values for the location were the average of the 9 Landsat pixels. However, some of the plots we received (<2%) from other ground sample networks did have a polygon boundary. For these, we used average of all the Landsat pixels intersected by the polygon as the sample value. Some plots (<1%) were in the form of measurements along transects. These transects were divided into 5 sections. We treated each section as one sample

and used the average of all Landsat pixels intersected by that section of the transect as the sample value. We then used a 1km × 1km grid for all the transects to reduce the number of samples in each grid cell to 1.

At many PSP sites, there were multiple sample plots of the same size inside a larger boundary (typically 200m × 200m). In addition, many other samples (for example, samples from Bonanza Creek LTER) were closer to each other than 1 km. Such nearby samples had high spatial autocorrelation in the Landsat spectral bands and indices. We used a gridded approach to reduce the number of such samples leaving only one sample in a 1km × 1km grid cell. This step was performed before dividing samples into training and validation sets, reducing spatial auto-correlation effects across training samples and validation samples.

We did not use the PSPs that were disturbed in this study. The remaining PSPs were located more than 100m away from roads or human caused disturbances. This was done to ensure the 30m Landsat pixels in the 3 × 3 grid around the PSP do not capture roads or other human disturbances. In addition, the establishment procedure of PSPs from Canada did not include small trees (<1.5m)[5].

1.8 Deciduous fraction and tree canopy cover

Deciduous fraction depicts the fraction of trees that are deciduous within a plot. Since deciduous and evergreen plant composition types are mutually exclusive and collectively exhaustive in the NA boreal domain, low deciduous fraction implies high evergreen dominance in a pixel. While deciduous fraction is not a categorical depiction of canopy cover, it provides an estimate of vegetation composition or dominance. Therefore, a pixel can have a deciduous fraction of 1, on a

scale of 0 to 1, even if the vegetation is sparse (for example, 50% tree canopy cover) if all the trees inside that pixel are deciduous. On the other hand, the tree canopy cover product represents the percentage of a Landsat pixel that is completely covered by tree canopy. However, since this product is also derived solely from multispectral data, it is susceptible to influence from large or high-density shrubs and understory vegetation that have the same spectral signal as tree canopy. It should be noted that since tree canopy cover and deciduous fraction layers were developed separately, these products are not influenced by each other and can be used independently.

1.9 Ground sample selection

We used permanent sample plots across Alaska and Canada from multiple studies and agencies in this study (Supplementary table 1 & figure 13). Ground samples for deciduous fractional cover were checked for spatial auto correlation and sample density across the output deciduous values. We used 1 km × 1 km grid to reduce the number of ground samples to 1 sample per 1 sq km grid cell. This reduced the overall sample sites from 27,494 to 22,566. In addition, we further divided the deciduous samples into eastern and western sample sets. These samples sets were still unbalanced and contained a large number of samples for all evergreen (deciduous fraction = 0) and all deciduous (deciduous fraction = 1). We therefore divided each 0 – 1 range into 10 bins and resampled all bins. To under-sample each bin, we used the 25th percentile of the number of samples in each bin. This method reduced the sample value bias at the ends of 0 – 1 range by providing a near-uniform distribution of sample density of the deciduous fraction values. The total number of samples was reduced by 56% using this method, with 61% and 48% reductions in samples for the eastern and western parts of boreal domain, respectively. Basal area from PSP data was used to assign a deciduous fraction value at each sample site. The inventory data do not

include cover estimates, but basal area-derived deciduous fractional cover indicative of deciduous leaf area and tree cover.

1.10 Method design

We used nominal years 2000 and 2015 to identify differences in deciduous fraction, tree canopy cover, predicted albedo, and radiative forcing. These two years provided adequate spatial coverage to detect changes across Alaska and Canada. For both 2000 and 2015 nominal years, we extracted average deciduous fraction, tree canopy cover, and surface albedo values inside fire polygons to assess changes due to fire in these three layers. The difference between each of the three layers for each polygon was used to assess the progressive change with the date of fire occurrence. For fires that occur between 2000 and 2015, the changes in deciduous fraction, tree cover, and albedo are large compared with gradual changes for fires that occurred before 2000. The fires that occur before 2000 are purely the 15-year difference inside the fire polygon between the two layers. In contrast, the fires that occur between 2000 and 2015 include the difference or loss in vegetation that the fire caused in addition to any vegetation growth that occurred post-fire. Since the difference in pre- and post-fire vegetation is typically quite large, the changes in deciduous fraction, tree cover and albedo, are quite large during that period. This is also evident in the radiative forcing cooling effects observed in that time-period.

1.11 Blue-sky albedo

Boreal North America experiences periodic and, in many cases, perennial snow and ice. This environment necessitates the consideration of incident radiation from both black-sky and white-sky surface albedo scenarios. Blue-sky albedo combines these two scenarios in a model that

better represents the albedo in such high latitude environments. We used fixed seasons across the North American boreal domain to compare spatial patterns in albedo.

We used predicted blue-sky albedo to compute radiative forcing using the CESM-CAM5 kernels. Specifically, we used albedo-specific radiative forcing kernels from the Community Atmosphere Model 5 (CAM5) in the Community Earth System Model (CESM) to calculate seasonal forcing. To do so, we followed the existing methods[6] and first estimated a change in albedo between the two time periods. These pixel-level changes were then multiplied by the spatially explicit kernels from [7], which were spatially downscaled to match the 30m resolution of our product. Our aim was to assess variability in radiative forcing as caused by deciduous fraction and tree canopy cover. While our random forest model cannot explain all the variability in blue-sky albedo prediction based solely on the deciduous fraction and tree canopy cover, we were able to identify the uncertainties in predicted albedo and in predicted radiative forcings due to deciduous fraction and tree canopy cover. We then analyzed changes in predicted mean blue-sky albedo for fire perimeters for spring, summer, and fall seasons across the boreal domain.

1.12 Significant change

To assess significant per-pixel change across the boreal domain from 2000 to 2015, we used a paired t-test since we used the same set of random forest models across different epochs. The t-test assesses significant difference between the random forest model outputs between the two epochs. The null hypothesis we used for the test stated that there is no statistical difference between the random forest output of 2000 and 2015. Pixels with $p\text{-value} < 0.05$ were determined to have significant change from 2000 to 2015.

To do this, we used the mean and standard deviation of the decision tree outputs from the random forest model. The test was performed separately for the west and east divisions and the regions with 3 season and 1 season models in the boreal domain. We used the uncertainty layers from both epochs as the standard deviation and difference between 2000 and 2015 deciduous fraction as mean of sample differences. We were able to identify regions of positive and negative significant and insignificant changes in the boreal domain (Supplementary table 6 & 7; Supplementary figure 14 & 15).

Appendix 2: Tables

Supplementary table 1: Ground samples used from multiple site networks in the North American boreal domain. All sample locations were combined into one large dataset for model training and validation.

**Sites**	**Location**	**Number of samples**
Canadian permanent sample plots	Canada	26,831
Northwest Territories chronosequence data	Northwest Territories, Canada	263
Canada National Forest Inventory	Canada	195
Cooperative Alaska Forest Inventory	Alaska, USA	142
Bonanza Creek LTER	Alaska, USA	63
Total		27,494

Supplementary table 2: Average change in forest deciduous fraction and areal extent of changes from 2000 to 2015 across treed (tree canopy cover ≥ 25%) boreal regions and ecoregions in North America. Deciduous fraction ranges from 0 to 1. Eastern Canada includes Ontario, Quebec, Newfoundland, and Labrador, while Western Canada includes Yukon, Northwest Territories, Nunavut, British Colombia, Alberta, Saskatchewan, and Manitoba. The areas in parenthesis show significant increase or decrease for the boreal region or ecoregion.

**Domain**	**Average change in deciduous fraction**	**Area of increase in deciduous fraction (Mha)**	**Area of decrease in deciduous fraction (Mha)**
<u>Region</u>			
Eastern Canada	-0.023 ± 0.015	65.8 (52.0)	89.6 (73.9)
Western Canada	-0.019 ± 0.012	84.4 (69.3)	116.8 (93.8)
Alaska	-0.058 ± 0.035	16.5 (15.4)	34.7 (33.1)
<u>Ecoregion</u>			
Taiga Plain	-0.010 ± 0.008	19.7 (16.6)	24.5 (19.3)
Taiga Shield West	0.009 ± 0.005	9.2 (9.1)	11.0 (7.8)
Taiga Shield East	-0.005 ± 0.004	16.4 (12.7)	18.4 (14.2)
Boreal Shield East	-0.033 ± 0.021	32.3 (25.4)	40.3 (33.9)
Boreal Shield West	-0.014 ± 0.01	28.4 (23.0)	34.4 (26.9)
Boreal Plain	-0.014 ± 0.008	16.2 (16.2)	40.9 (24.2)
Taiga Cordillera	-0.057 ± 0.041	19.1 (13.5)	23.8 (24.7)
Boreal Cordillera	-0.055 ± 0.042	15.7 (12.9)	29.3 (23.3)
Hudson Plain	-0.019 ± 0.009	9.6 (6.7)	13.9 (10.2)

Supplementary table 3: Image composites for early spring, mid-summer, and fall seasons used to identify key differences in deciduous and evergreen green-up amplitudes. Nominal 1992 Landsat data did not have complete coverage for Alaska.

Year range	Julian dates	Season	Data	Region
1987 – 1997	55 - 165	Spring	Landsat 5	Canada
(nominal 1992)	180 - 240	Summer		
	255 - 330	Fall		
1998 – 2002	55 - 165	Spring	Landsat 5 & 7	Canada, Alaska
(nominal 2000)	180 - 240	Summer		
	255 - 330	Fall		
2003 – 2007	55 - 165	Spring	Landsat 5 & 7	Canada, Alaska
(nominal 2005)	180 - 240	Summer		
	255 - 330	Fall		
2008 – 2012	55 - 165	Spring	Landsat 5 & 7	Canada, Alaska
(nominal 2010)	180 - 240	Summer		
	255 - 330	Fall		
2013 – 2018	55 - 165	Spring	Landsat 7 & 8	Canada, Alaska
(nominal 2015)	180 - 240	Summer		
	255 - 330	Fall		

Supplementary table 4: Random Forest model parameters for deciduous fraction and tree canopy cover used in multi-dimensional grid search for optimal parameters. The RMSE values of deciduous fraction and tree cover models are for output values in the range of 0 – 1 and 0 – 100, respectively.

Parameter	Deciduous fraction				Tree canopy cover	
	Western Boreal		Eastern Boreal			
	3-season model	1-season model	3-season model	1-season model	3-season model	1-season model
n_estimators	800	800	1200	1200	2000	1200
max_features	4	2	4	2	12	8
min_sample_split	2	2	2	2	4	12
min_sample_leaf	1	1	1	1	2	4
max_depth	None	None	None	None	24	16
r-squared	0.71	0.56	0.54	0.48	0.61	0.52
RMSE	0.19	0.09	0.21	0.24	21	23

Supplementary table 5: Random Forest model parameters for spring, summer and fall blue-sky albedo models using deciduous fraction and tree canopy cover as inputs.

Parameter	Albedo		
	**Spring**	**Summer**	**Fall**
N estimators	2000	1800	1800
Max features	2	2	2
Min sample split	6	4	4
Min sample leaf	1	1	1
Max depth	None	None	None
RMSE	0.18	0.06	0.15
r-squared	0.37	0.46	0.28

Supplementary table 6: Comparison of deciduous fraction and tree canopy cover random forest models versus linear regression models. Note that tree canopy cover RMSE ranged from 0 – 100 in contrast with deciduous fraction which ranged from 0 – 1.

			Random forest model		Linear model	
	**Region**	**Features**	**R-squared**	**RMSE**	**R-squared**	**RMSE**
Deciduous fraction	Eastern Boreal	3-season	0.54	0.21	0.29	0.08
		1-season	0.48	0.24	0.28	0.12
	Western Boreal	3-season	0.71	0.19	0.44	0.05
		1-season	0.56	0.09	0.31	0.09
Tree canopy cover		3-season	0.61	21	0.21	18
		1-season	0.52	23	0.15	16

Supplementary table 7: Significant versus insignificant change in tree covered fire polygons across boreal ecozones. Tree covered pixels were decided by max tree cover > 0.25 across all observed epochs (2000 – 2015).

	Positive change (Mha)		Negative change (Mha)	
**Zone**	**Significant**	**Insignificant**	**Significant**	**Insignificant**
Taiga Plain	5.84	0.68	5.53	0.69
Taiga Shield West	4.05	0.65	2.97	0.61
Taiga Shield East	1.26	0.17	1.02	0.17
Boreal Shield East	2.96	0.22	1.98	0.21
Boreal Shield West	9.38	1.58	9.12	1.59
Boreal Plain	5.95	0.80	7.28	0.87
Taiga Cordillera	6.19	0.67	8.04	0.74
Boreal Cordillera	3.87	0.37	4.35	0.40
Hudson Plain	0.81	0.11	0.66	0.13

Supplementary table 8: Significant versus insignificant change in tree covered areas across boreal ecozones in million hectares (Mha). Tree covered pixels were decided by max tree cover > 0.25 across all observed epochs (2000 – 2015).

	Positive change (Mha)		Negative change (Mha)	
**Zone**	**Significant**	**Insignificant**	**Significant**	**Insignificant**
Taiga Plain	16.44	2.68	19.34	2.85
Taiga Shield West	8.88	1.92	7.82	1.91
Taiga Shield East	12.44	2.91	14.22	3.21
Boreal Shield East	24.86	4.00	33.92	4.31
Boreal Shield West	22.44	4.39	26.82	4.89
Boreal Plain	18.70	2.84	24.05	3.16
Taiga Cordillera	13.16	2.02	24.66	2.53
Boreal Cordillera	12.64	1.95	23.30	2.36
Hudson Plain	6.63	2.12	10.23	2.83

Supplementary table 9: Percentage by area where 3-season and 1-season models were used for the entire Canada and Alaska region and for the boreal domain. The no model area includes water bodies and perennial snow or ice regions.

	**Canada & Alaska**			**Boreal domain**		
	**3-Season model**	**1-season model**	**No model**	**3-Season model**	**1-season model**	**No model**
1992	70.1%	21.8%	8.1%	83.6%	12.2%	4.2%
2000	74.9%	18.9%	6.1%	87.9%	7.8%	4.4%
2005	77.9%	16.4%	5.8%	90.0%	6.3%	3.7%
2010	75.0%	18.4%	6.6%	89.4%	5.7%	4.8%
2015	81.2%	14.3%	4.5%	91.2%	5.9%	2.9%

Appendix 3: Figures

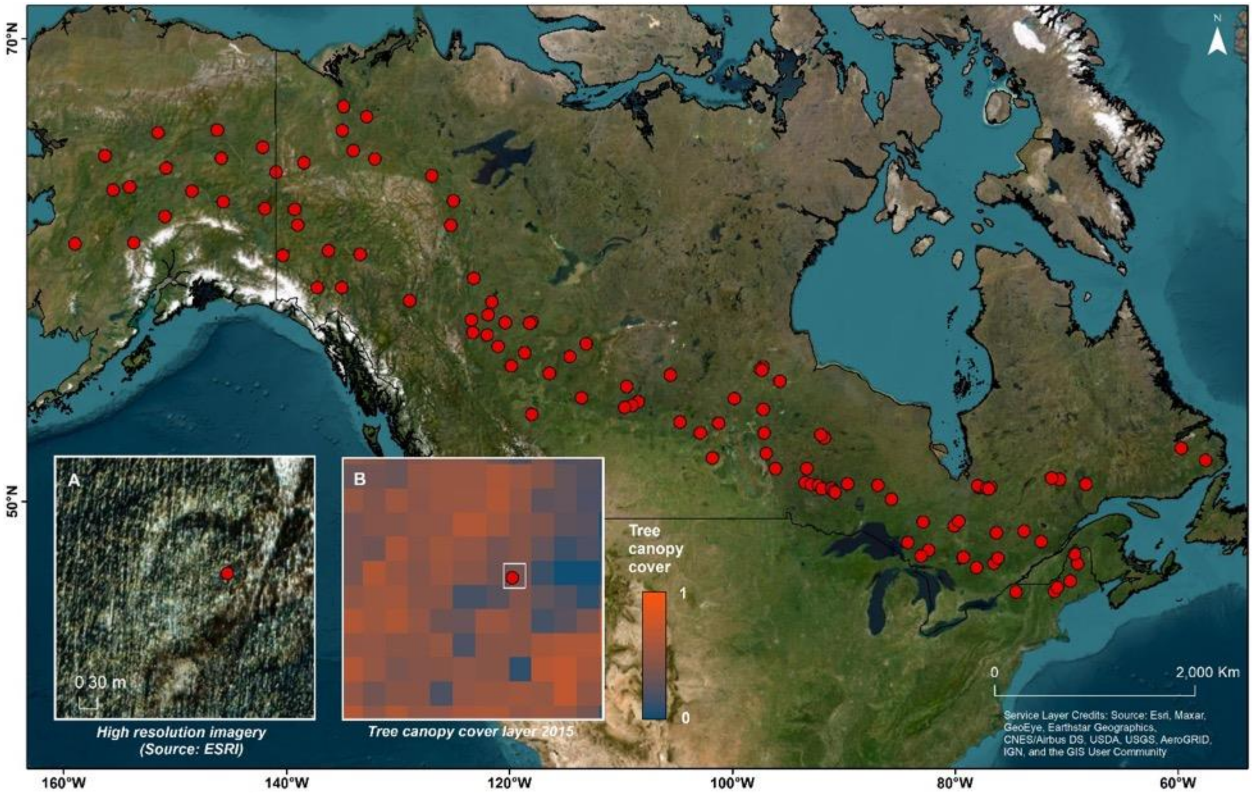

Supplementary figure 1: Visual assessment locations (n = 110) between high-resolution imagery mosaics from GeoEye-1 and WorldView-3 satellite data at 1.65m spatial resolution provided by ESRI via their customer user agreement **(A)** and tree canopy cover pixels at 30m spatial resolution **(B)** for the same nominal year across the boreal domain to determine per pixel tree cover threshold of 25%. The pixel marked in panel **(B)** has a tree canopy cover value of 0.25.

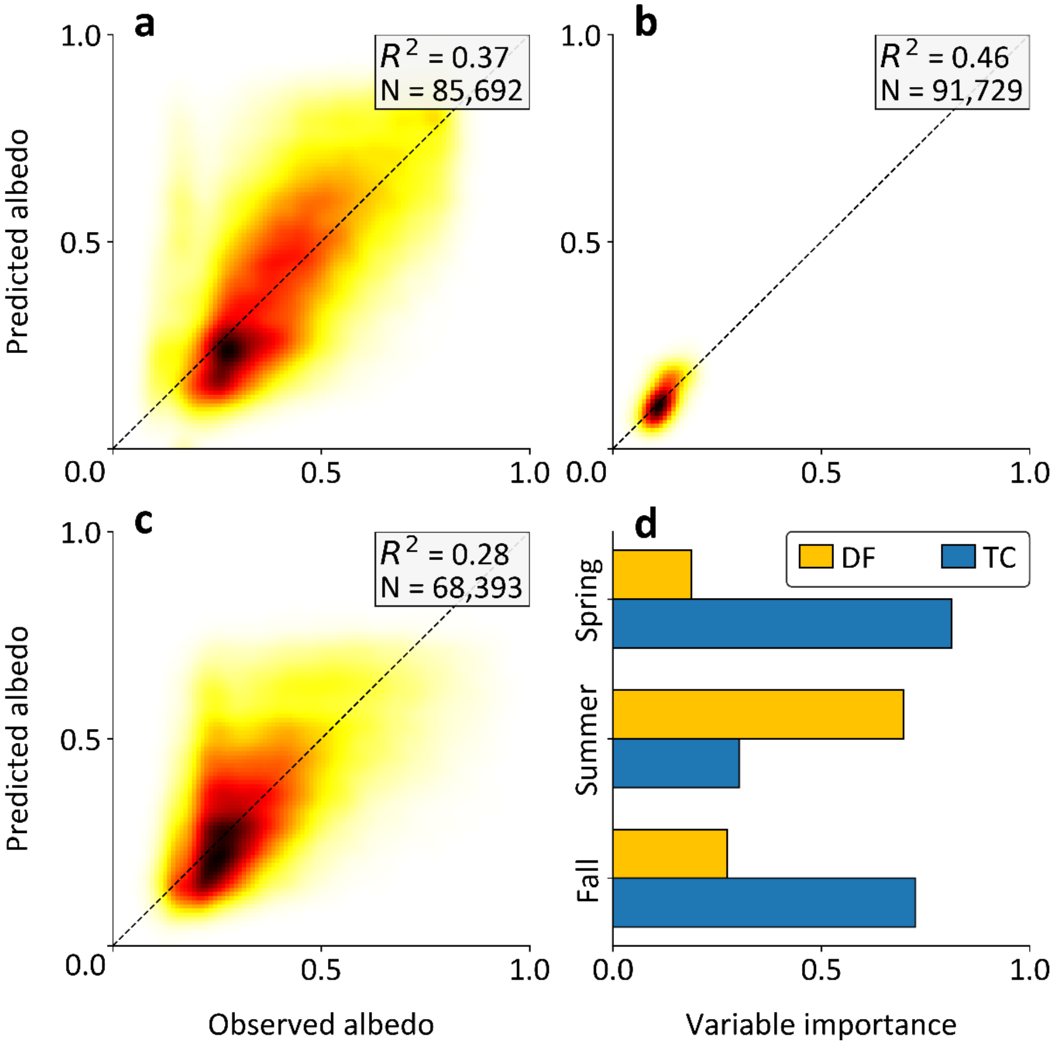

Supplementary figure 2: Performance of **(a)** spring, **(b)** summer, and **(c)** fall random forest models and **(d)** variable importance plot for surface albedo using deciduous fraction (DF) and tree cover (TC) as input layers. Evaluation performed using cross-validation.

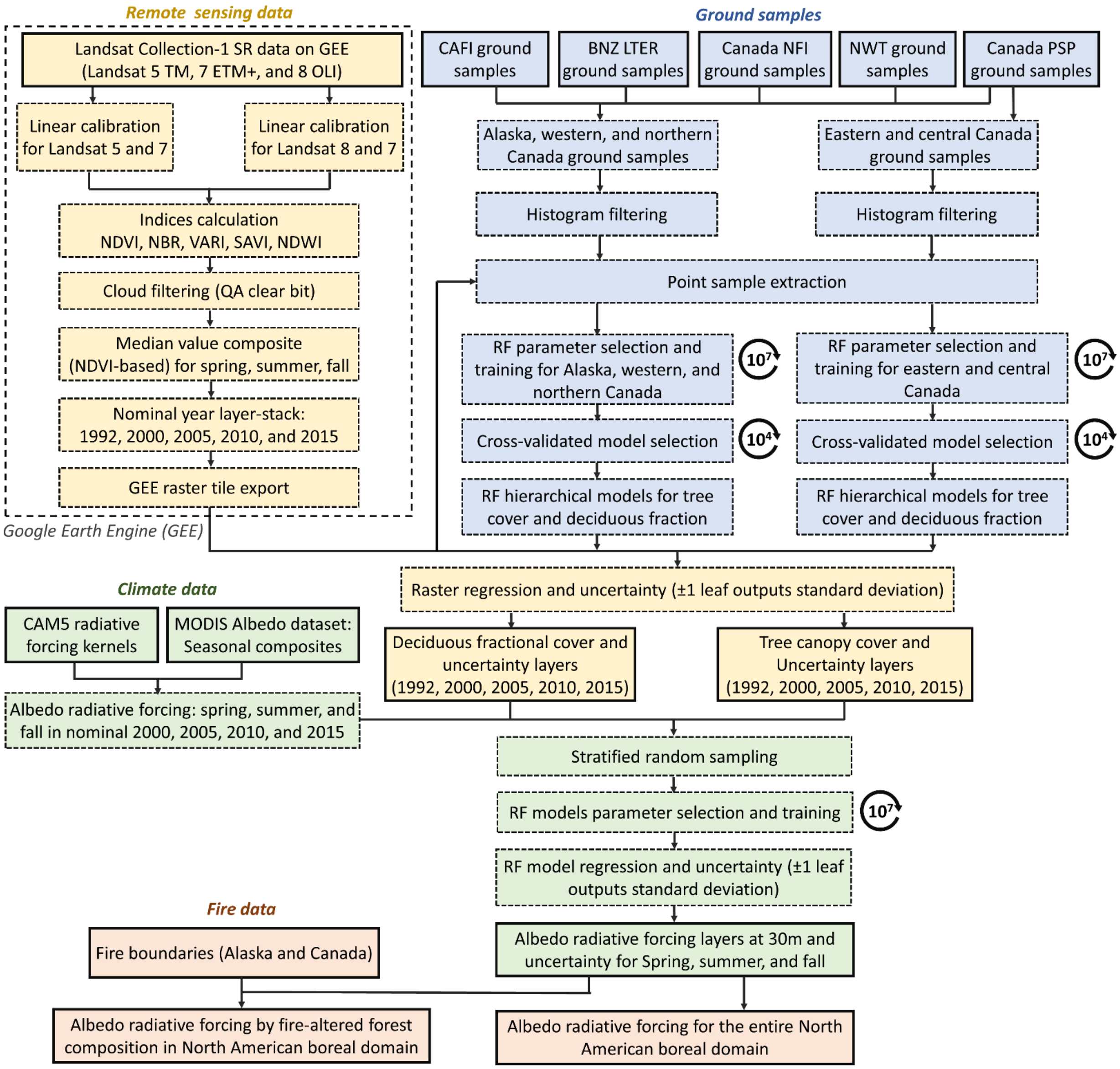

Supplementary figure 3: Overall workflow for preparing deciduous fraction layers, tree canopy cover layers, and surface albedo layers for nominal years 1992, 2000, 2005, 2010, and 2015 across boreal North America. Here sold line blocks indicate input or output layers. Dashed line blocks are intermediate layers.

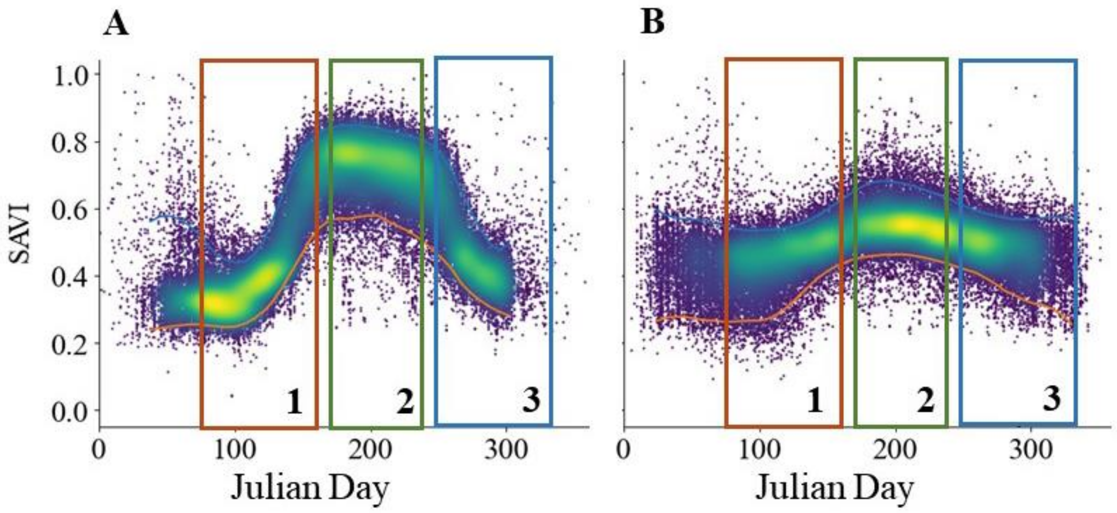

Supplementary figure 4: Seasonal soil adjusted vegetation index (SAVI) for **(A)** deciduous and **(B)** evergreen field samples sites for nominal years 1992, 2000, 2005, 2010, and 2015. Sample categories were defined here using deciduous fraction thresholds of greater than 0.75 for deciduous and less than 0.25 for evergreen. Windows shows spring (1), summer (2), and fall (3).

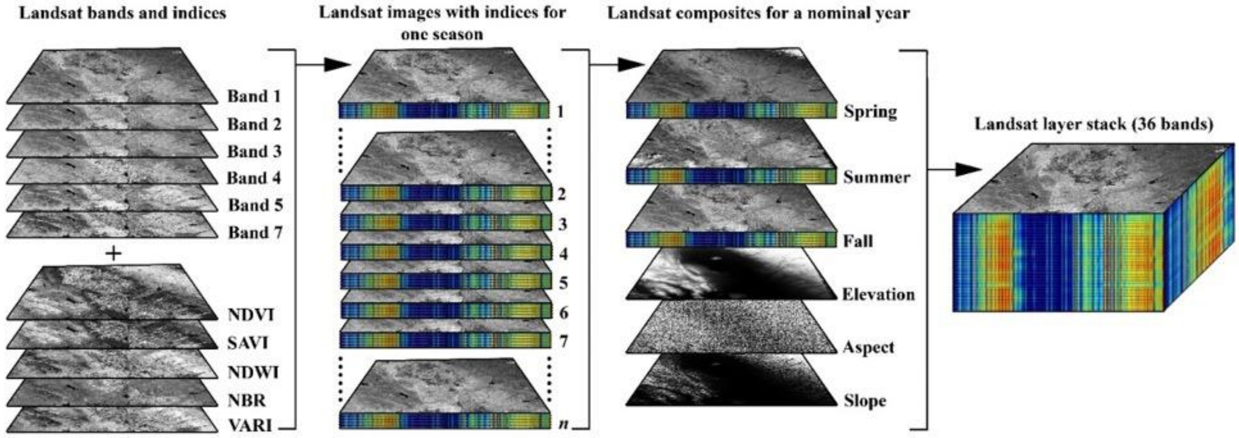

Supplementary figure 5: Landsat image stack used for modeling. Landsat image composites prepared using visible and NIR bands, as well as derived indices for spring, summer, and fall. Topographical layers were also added to the seasonal composites to produce a 36-band layer-stack.

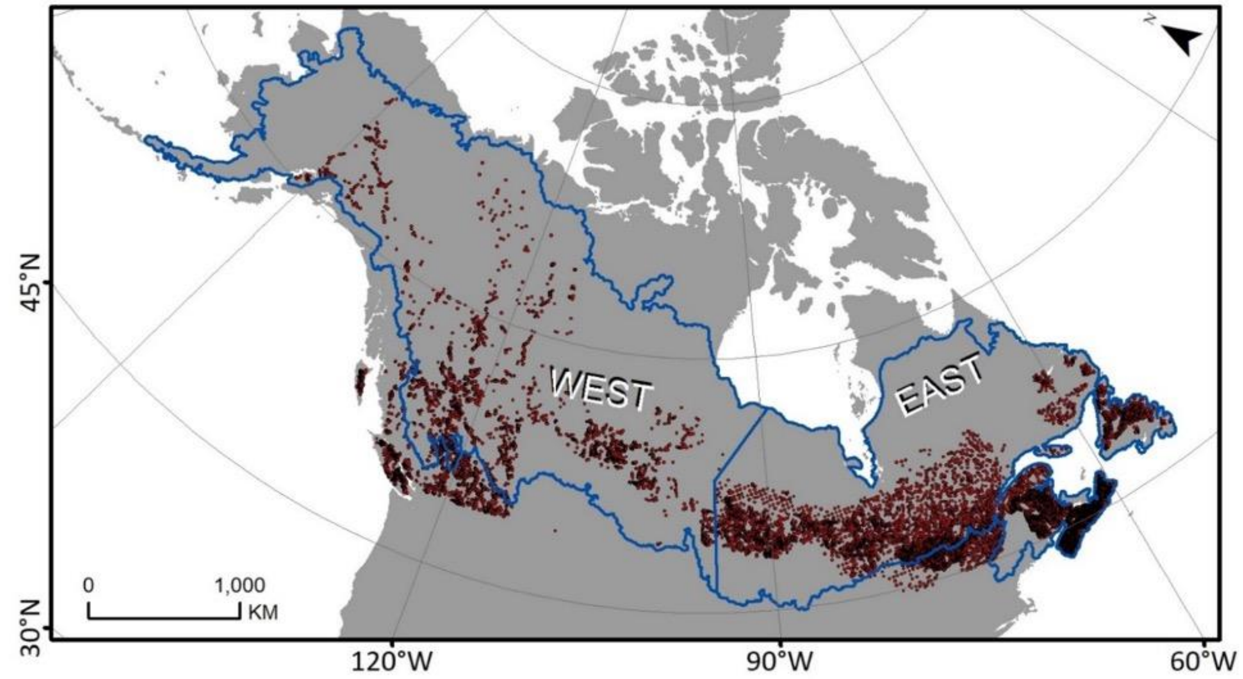

Supplementary figure 6: Ground samples used in deciduous fraction analysis divided into east and west regions.

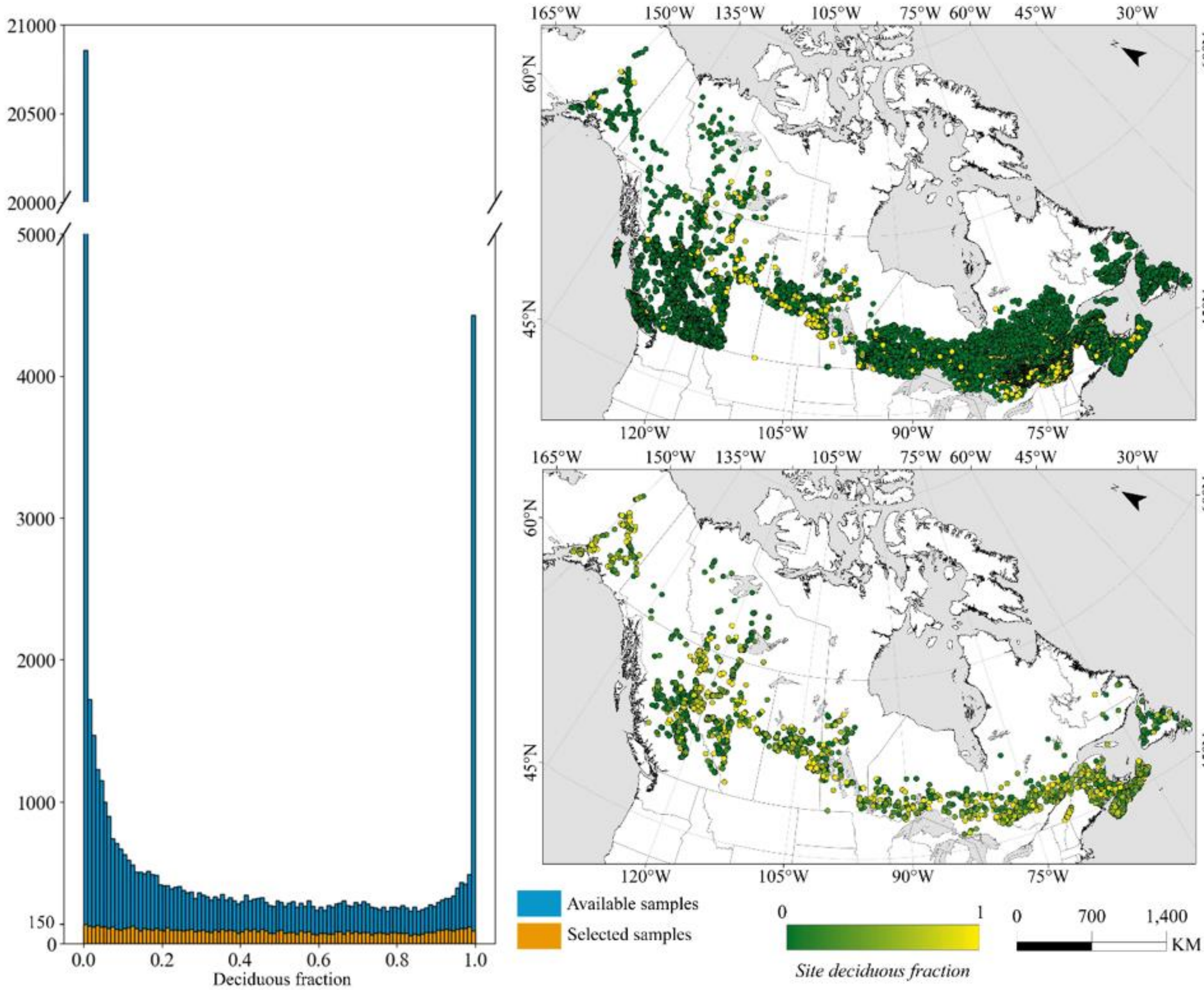

Supplementary figure 7: Resampling of ground samples by **(A)** binned under-sampling to create a near uniform distribution of deciduous fraction and reduction of sample size from **(B)** to **C**.

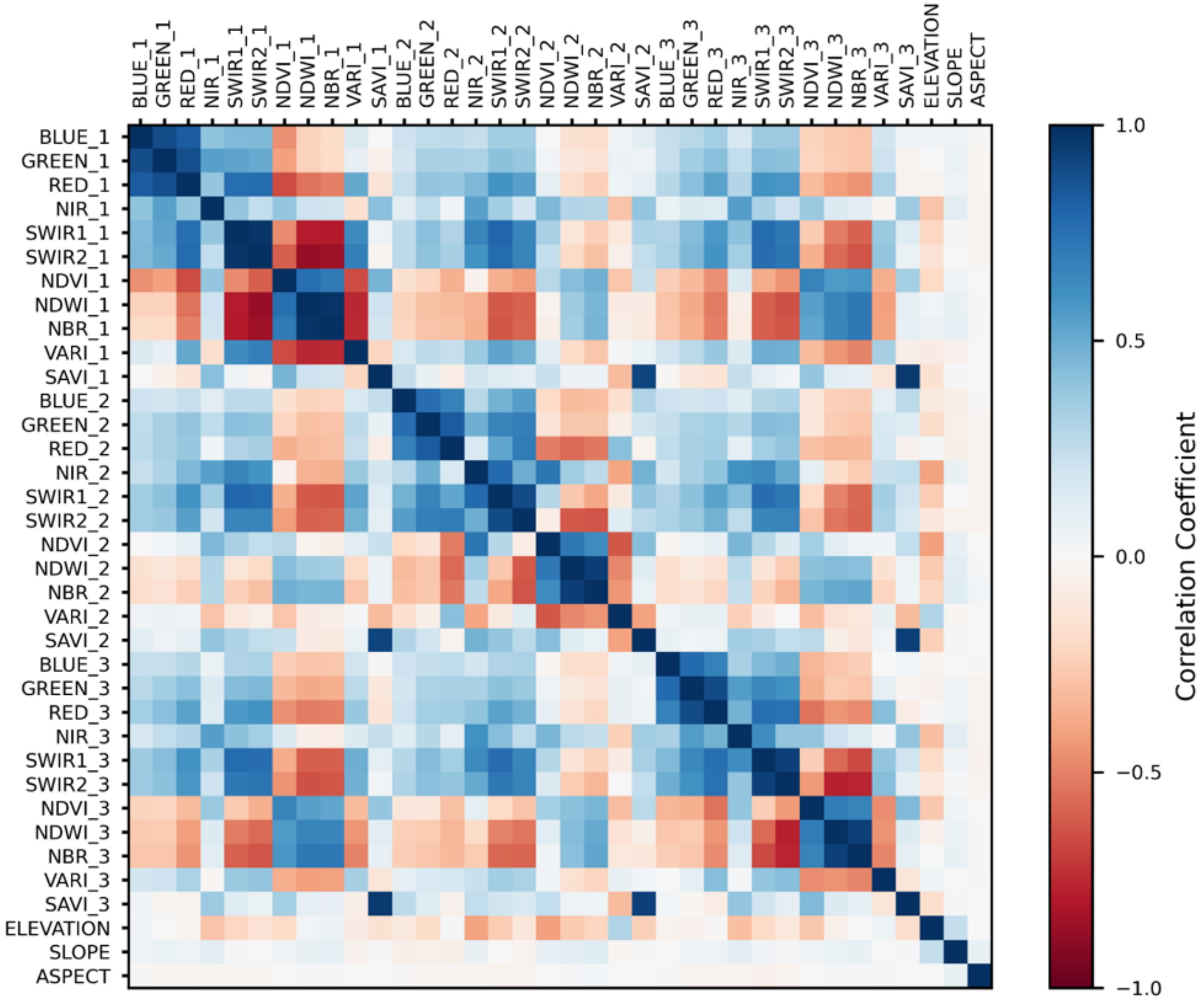

Supplementary figure 8: Correlation matrix between all Landsat bands and spectral indices derived using all the extracted samples across nominal years 1992, 2000, 2005, 2010, and 2015. Seasons include spring (1), summer (2), and fall (3).

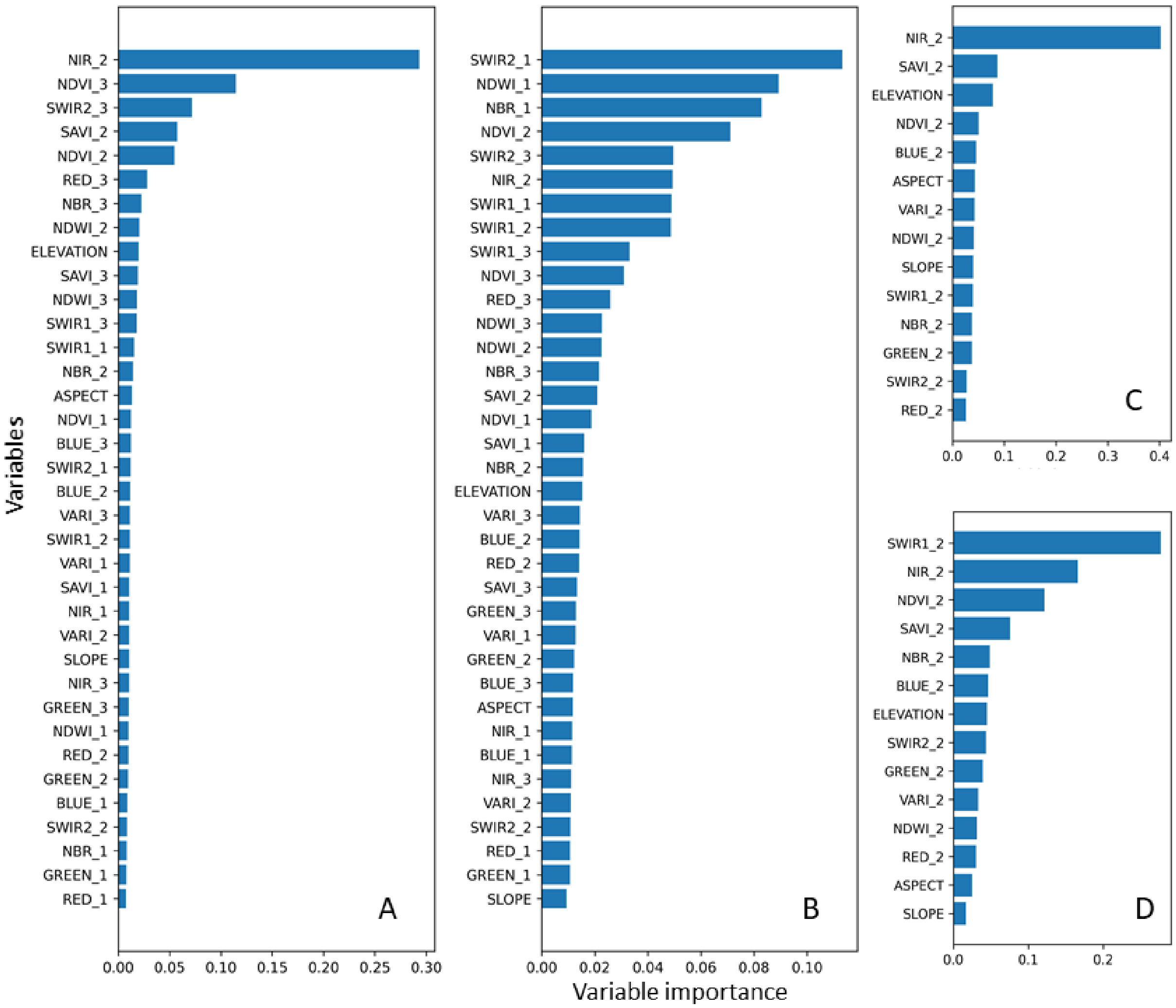

Supplementary figure 9: Deciduous fraction variable importance plots. (A) 3-season model for western zone, **(B)** 3-season model for eastern zone, **(C)** 1-season model for western zone, and **(D)** 1-season model for eastern zone.

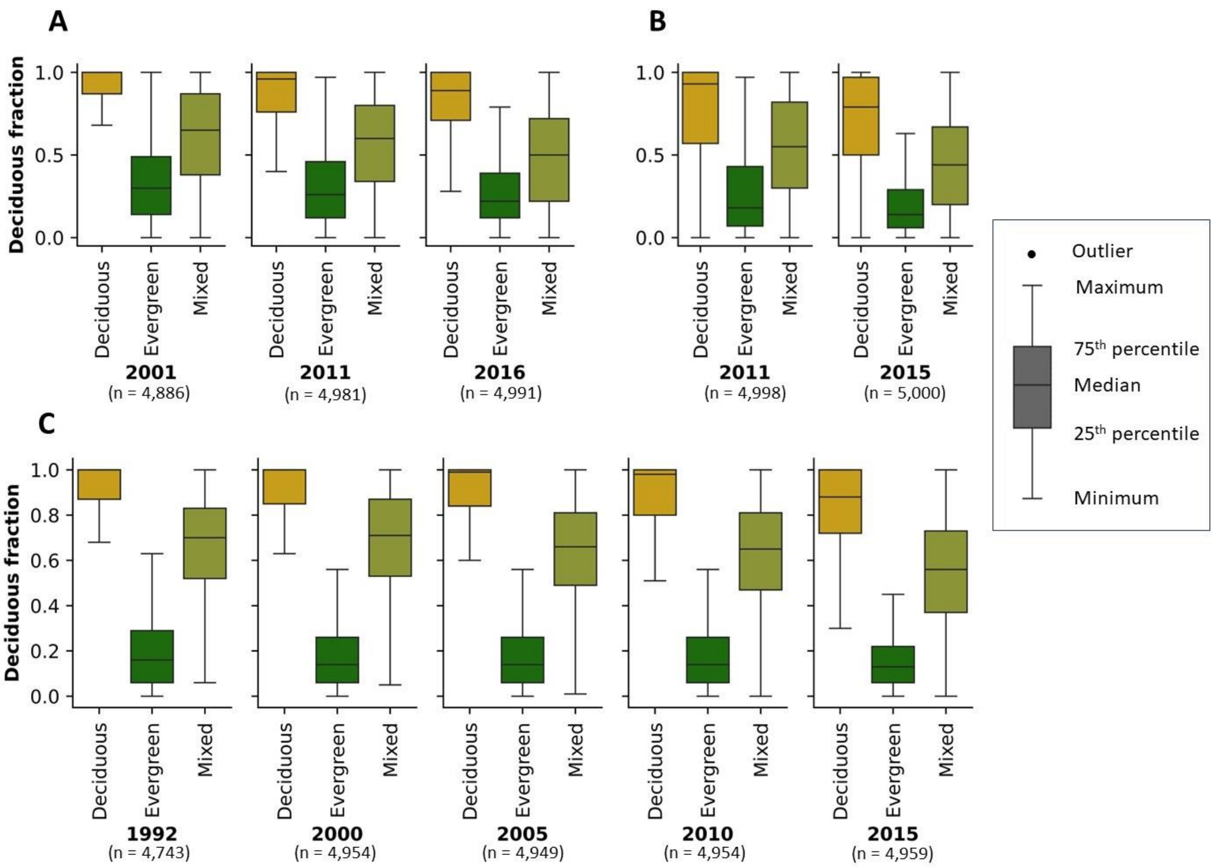

Supplementary figure 10: Deciduous fraction layers 1992 – 2015 compared with categorical landcover maps: A) Agriculture and agri-food Canada (AAFC) cropland layers, B) national land cover data (NLCD) for Alaska, and C) arctic boreal vulnerability experiment (ABoVE) annual dominant landcover layers.

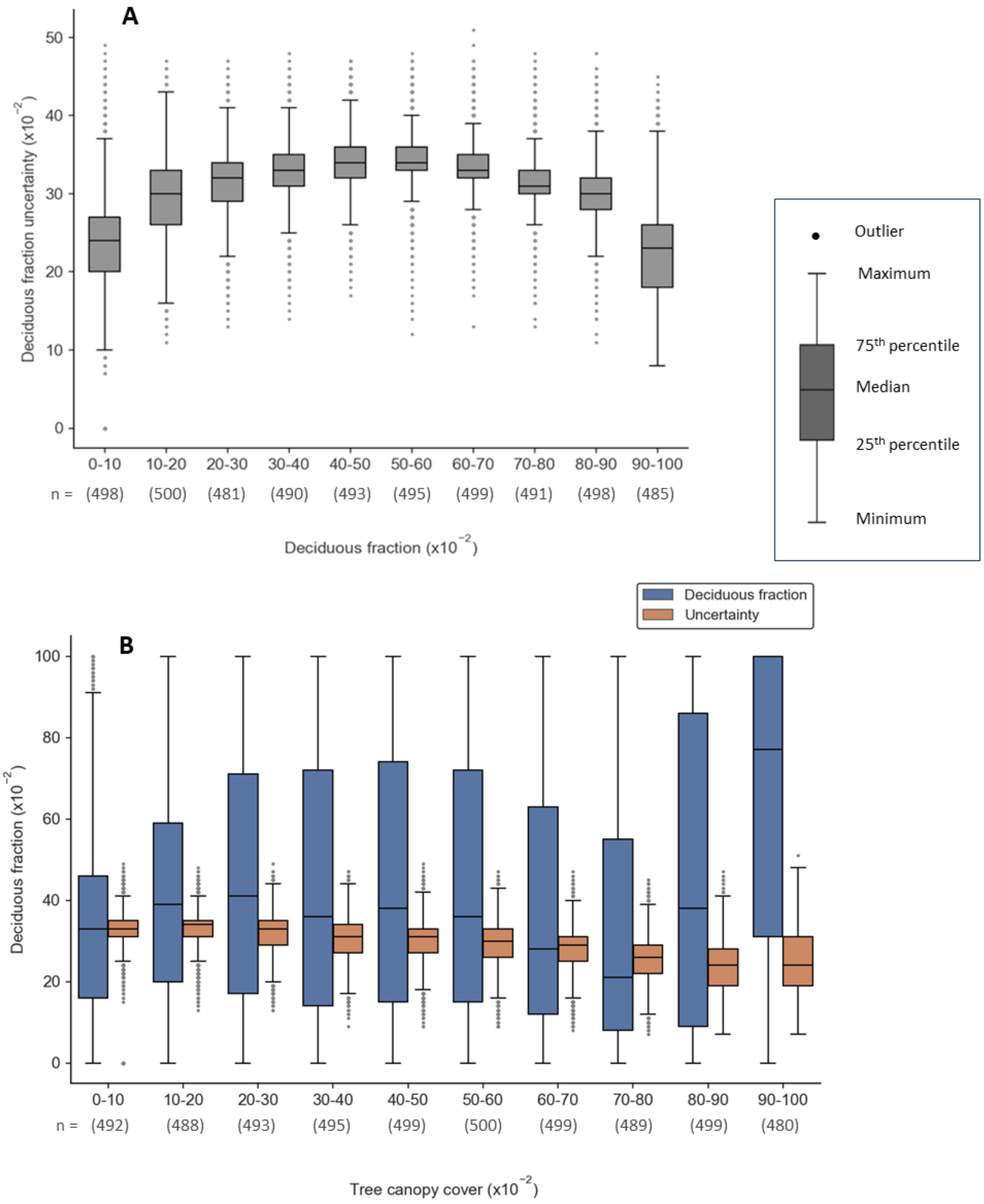

Supplementary figure 11: **(a)** Relationships between per-pixel deciduous fraction and its uncertainty, **(b)** per-pixel tree canopy cover and deciduous fraction. The samples for each boxplot (n~500) were obtained randomly for each interval from the respective layers.

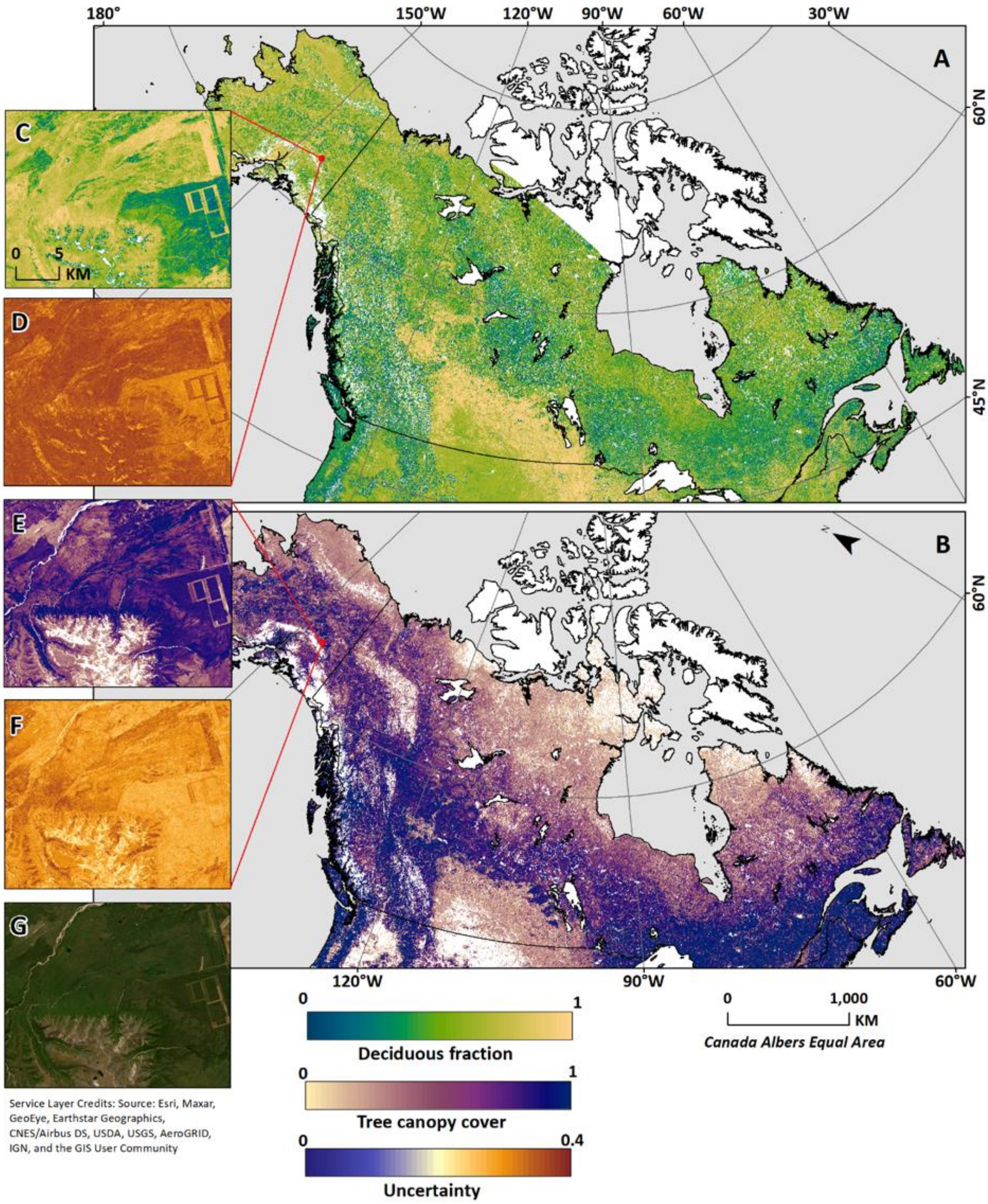

Supplementary figure 12: (A) Deciduous fraction and **(B)** tree canopy cover layer in 2015 for boreal North America. **(D)** The uncertainty map for deciduous fraction shows the absolute value of per-pixel uncertainty in deciduous fraction. Similarly, **(E)** tree canopy cover and **(F)** it's uncertainty. **(G)** High resolution imagery from Google Earth Engine is provided as reference.

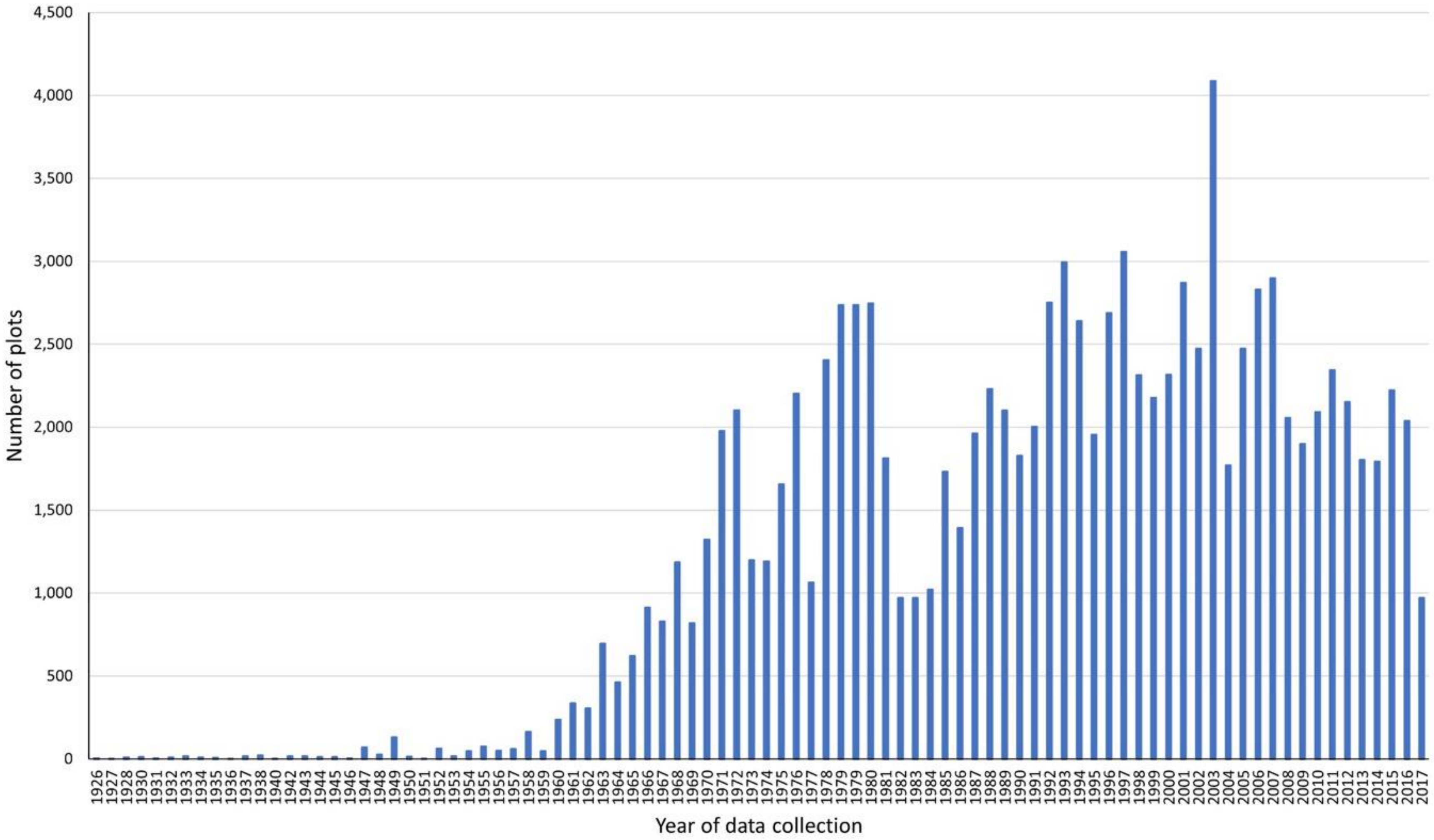

Supplementary figure 13: Number of multi-agency permanent sample plots (PSPs) data collected by year across Alaska and Canada. PSP data is typically collected at regular intervals after a sample plot is established.

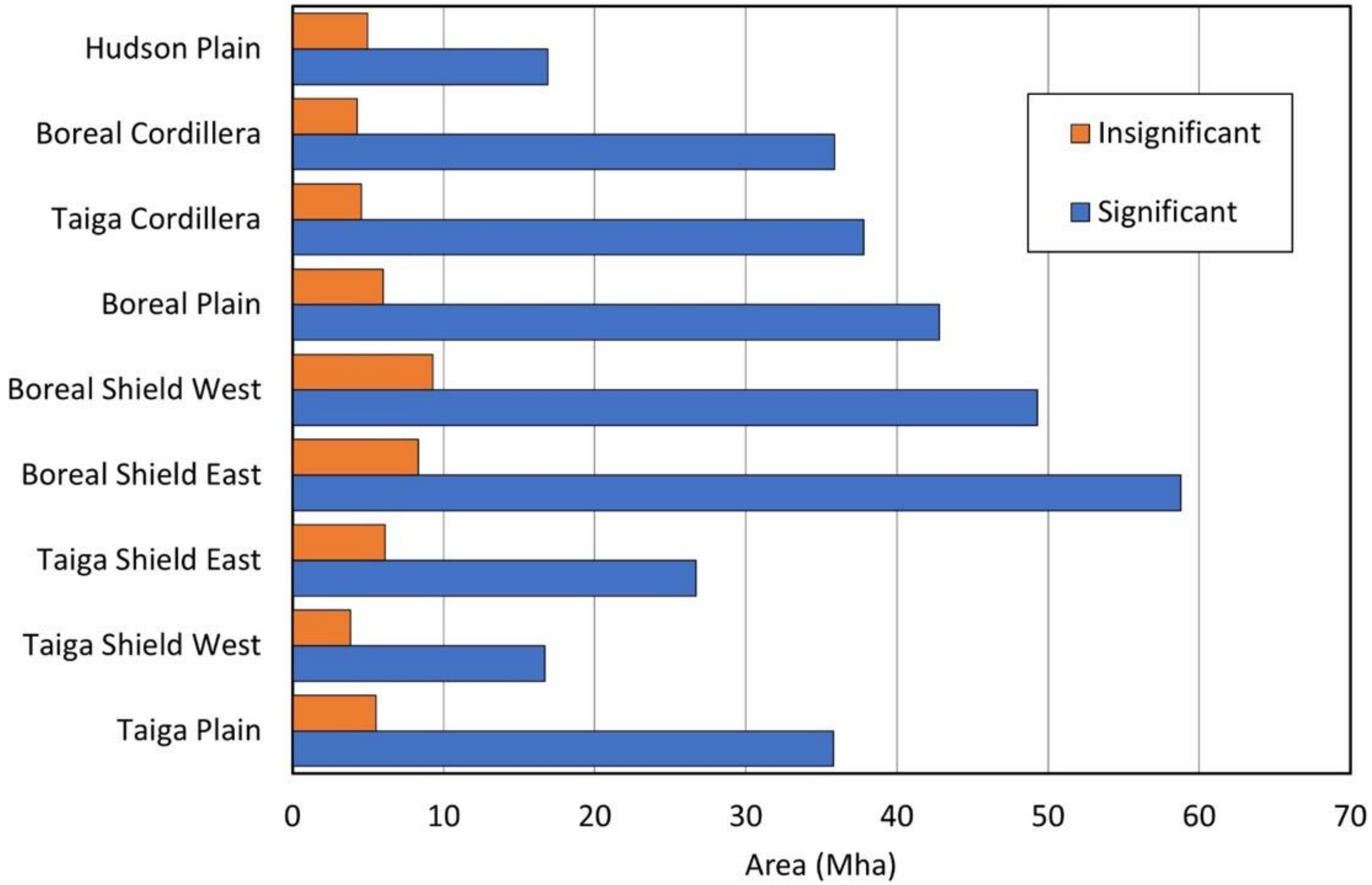

Supplementary figure 14: Area of significant versus insignificant change in deciduous fraction from 2000 to 2015 across ecoregions in the boreal domain. The unit of measurement is million hectares (Mha).

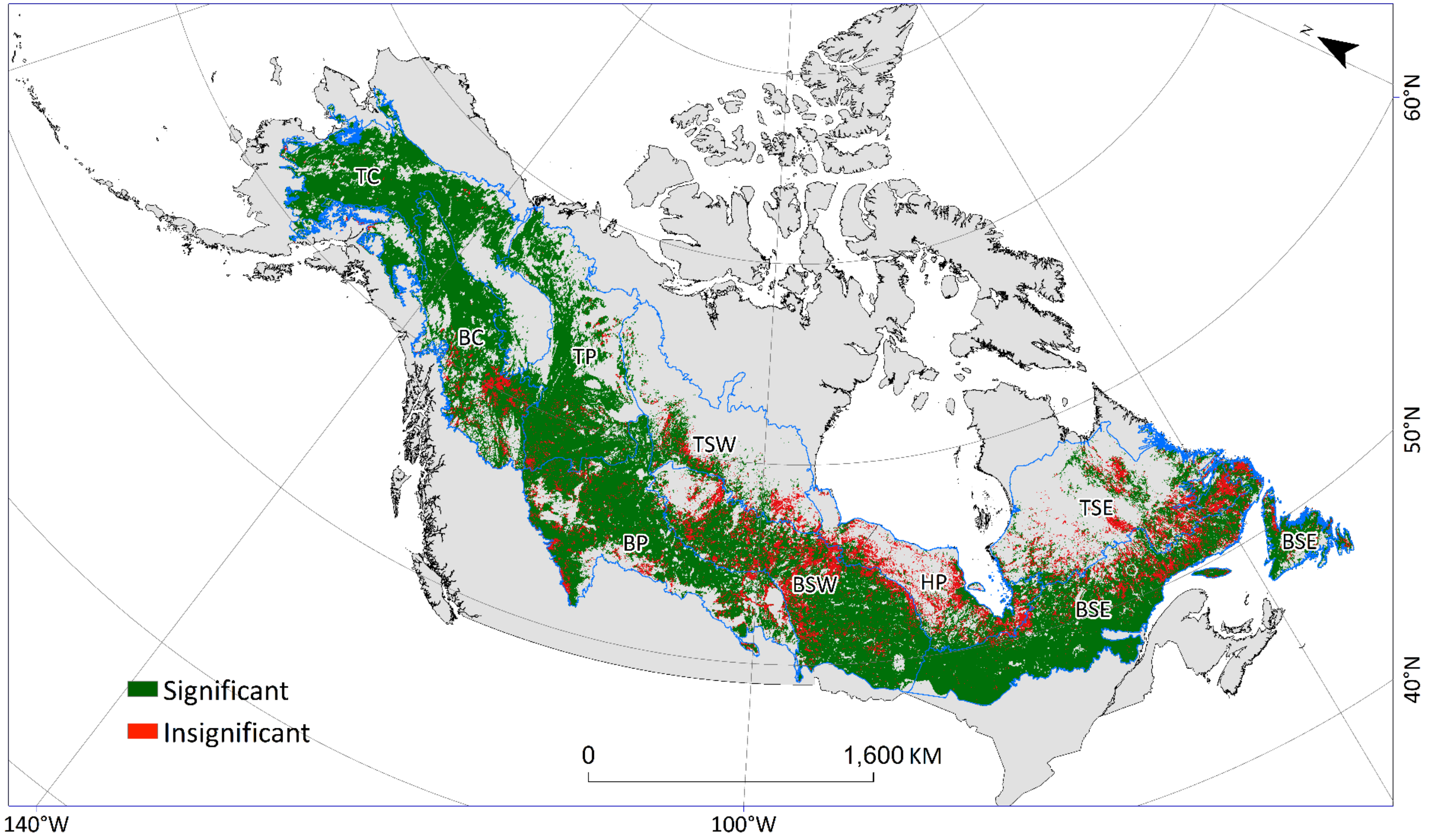

Supplementary figure 15: Map depicting significant and insignificant change in tree covered (per-pixel tree cover > 25%) deciduous forest in boreal domain ecoregions.

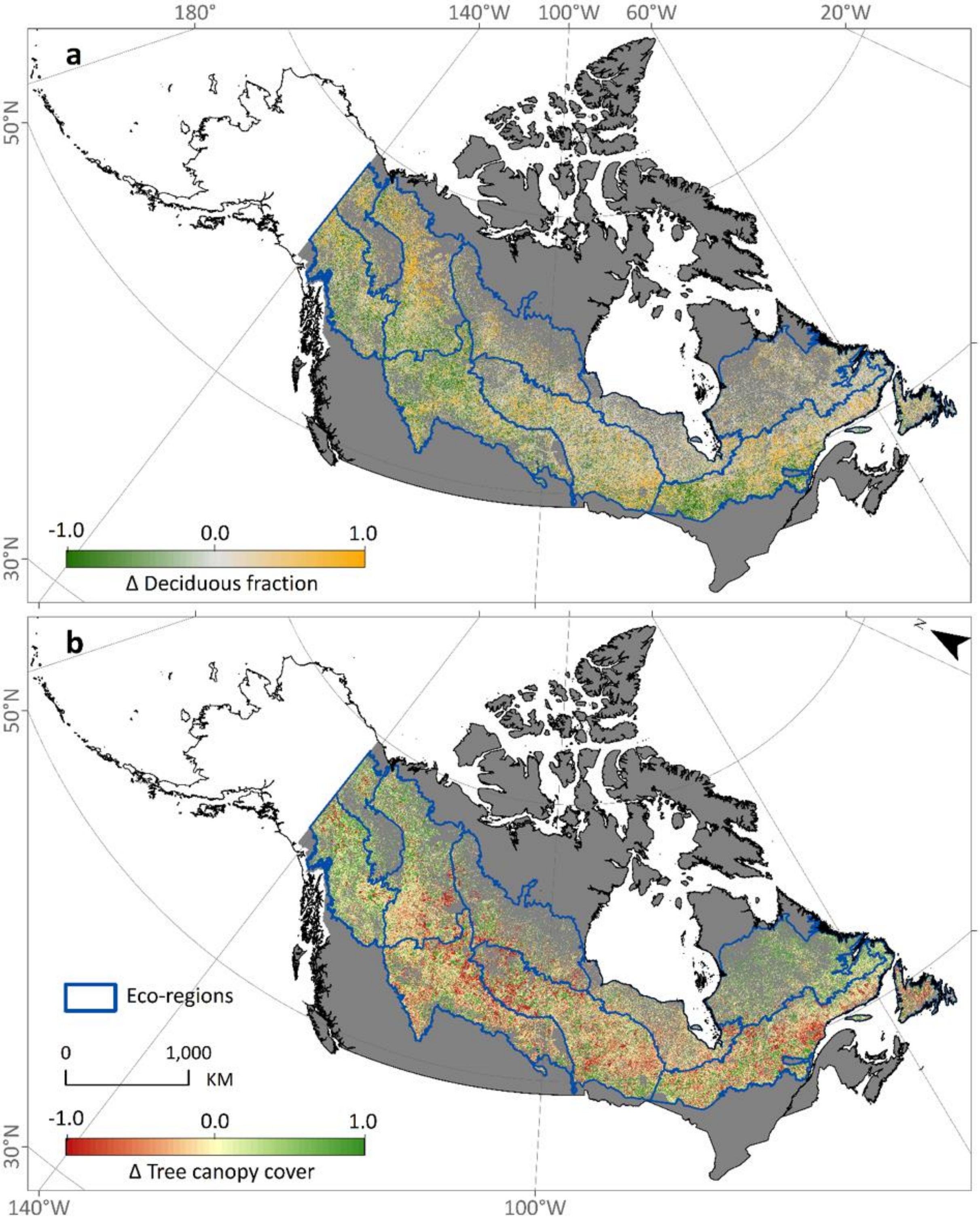

Supplementary figure 16: Change in deciduous fraction (**a**), and tree canopy cover (**b**), from 1992 – 2015 for the Boreal domain in Canada.

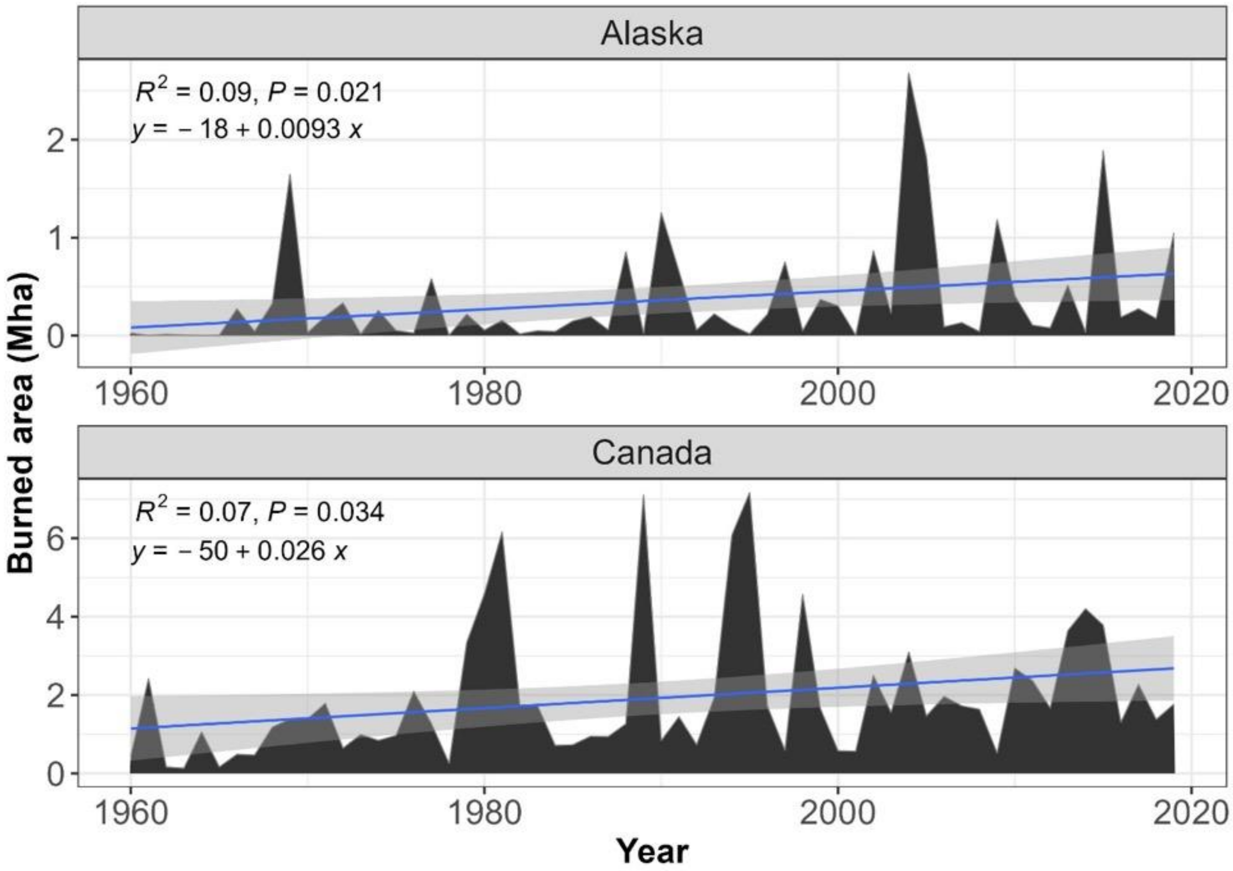

Supplementary figure 17: Annual burned area from 1960-2019 across Alaska and Canada. Units are in millions of hectares (Mha) burned per year. Blues lines depict best-fit linear regressions, while shaded bands depict 95% confidence intervals (n = 60 for each region). Burned area data from [8].

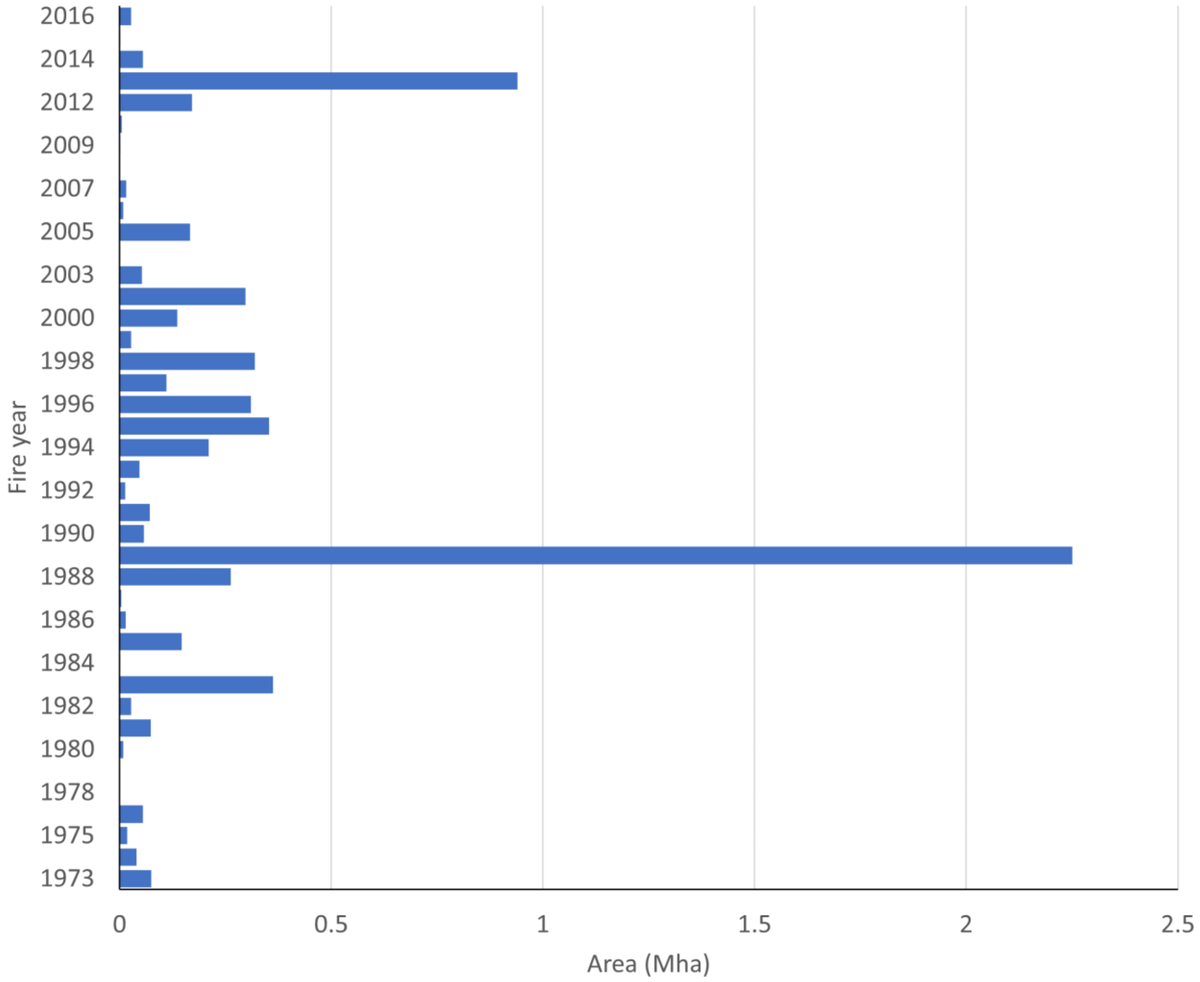

Supplementary figure 18: Distribution of area burned by year for ecoregion Taiga Shield East.

References:

1. Potapov P, Hansen MC, Laestadius L, Turubanova S, Yaroshenko A, Thies C, *et al.* The last frontiers of wilderness: Tracking loss of intact forest landscapes from 2000 to 2013. *Science Advances* 2017, **3**(1)**:** e1600821.

2. Hansen MC, Potapov PV, Moore R, Hancher M, Turubanova SA, Tyukavina A, *et al.* High-Resolution Global Maps of 21st-Century Forest Cover Change. *Science* 2013, **342**(6160)**:** 850-853.

3. Sulla-Menashe D, Friedl MA, Woodcock CE. Sources of bias and variability in long-term Landsat time series over Canadian boreal forests. *Remote Sensing of Environment* 2016, **177:** 206-219.

4. Roy DP, Kovalskyy V, Zhang HK, Vermote EF, Yan L, Kumar SS, *et al.* Characterization of Landsat-7 to Landsat-8 reflective wavelength and normalized difference vegetation index continuity. *Remote Sensing of Environment* 2016, **185:** 57-70.

5. Alberta Agriculture and Forestry. *Minimum Standards and Suggested Protocol and Priorities for Establishing and Measuring Permanent Sample Plots in Alberta, Technical Report T/605*: Edmonton, Alberta, 2015.

6. Potter S, Solvik K, Erb A, Goetz SJ, Johnstone JF, Mack MC, *et al.* Climate change decreases the cooling effect from postfire albedo in boreal North America. *Global change biology* 2020, **26**(3)**:** 1592-1607.

7. Pendergrass AG, Conley A, Vitt FM. Surface and top-of-atmosphere radiative feedback kernels for CESM-CAM5. *Earth System Science Data* 2018, **10**(1)**:** 317-324.

8. Phillips CA, Rogers BM, Elder M, Cooperdock S, Moubarak M, Randerson JT, *et al.* Escalating carbon emissions from North American boreal forest wildfires and the climate mitigation potential of fire management. *Science Advances* 2022, **8**(17)**:** eabl7161.
